# Graph Reinforcement Learning-Based Decision-Making Technology for Connected and Autonomous Vehicles: Framework, Review, and Future Trends

**DOI:** 10.3390/s23198229

**Published:** 2023-10-03

**Authors:** Qi Liu, Xueyuan Li, Yujie Tang, Xin Gao, Fan Yang, Zirui Li

**Affiliations:** 1 School of Mechanical Engineering, Beijing Institute of Technology, Beijing 100811, China; 3120195257@bit.edu.cn (Q.L.); 3120210298@bit.edu.cn (X.G.); 3120225230@bit.edu.cn (F.Y.); 3120195255@bit.edu.cn (Z.L.); 2 Faculty of Computer Science, Dalhousie University, Halifax, NS B3H 4R2, Canada; yujie.tang@dal.ca

**Keywords:** connected and autonomous vehicle, graph reinforcement learning, decision-making, mixed autonomy traffic

## Abstract

The proper functioning of connected and autonomous vehicles (CAVs) is crucial for the safety and efficiency of future intelligent transport systems. Meanwhile, transitioning to fully autonomous driving requires a long period of mixed autonomy traffic, including both CAVs and human-driven vehicles. Thus, collaborative decision-making technology for CAVs is essential to generate appropriate driving behaviors to enhance the safety and efficiency of mixed autonomy traffic. In recent years, deep reinforcement learning (DRL) methods have become an efficient way in solving decision-making problems. However, with the development of computing technology, graph reinforcement learning (GRL) methods have gradually demonstrated the large potential to further improve the decision-making performance of CAVs, especially in the area of accurately representing the mutual effects of vehicles and modeling dynamic traffic environments. To facilitate the development of GRL-based methods for autonomous driving, this paper proposes a review of GRL-based methods for the decision-making technologies of CAVs. Firstly, a generic GRL framework is proposed in the beginning to gain an overall understanding of the decision-making technology. Then, the GRL-based decision-making technologies are reviewed from the perspective of the construction methods of mixed autonomy traffic, methods for graph representation of the driving environment, and related works about graph neural networks (GNN) and DRL in the field of decision-making for autonomous driving. Moreover, validation methods are summarized to provide an efficient way to verify the performance of decision-making methods. Finally, challenges and future research directions of GRL-based decision-making methods are summarized.

## 1. Introduction

Intelligent transportation systems play an important role in both economic and social development, and connected and automated vehicles (CAVs) are an essential part of intelligent transportation systems [1]. Before fully autonomous driving is achieved, CAVs will operate for a certain period in mixed autonomy traffic, which includes both CAVs and human-driven vehicles (HVs) [2]. Therefore, the collaboration between CAVs and HVs and the communication between CAVs need to be carefully considered to ensure that CAVs can perform cooperative driving behaviors in mixed autonomy traffic [3]. Driving instructions of autonomous vehicles (AVs) are generated in decision-making systems. However, the simultaneous generation of driving instructions for multiple CAVs requires multi-agent decision-making systems. Therefore, designing a highly intelligent and reliable decision-making system for CAVs is crucial to generate reasonable driving behaviors in mixed autonomy traffic, which could improve the efficiency and safety of future intelligent transportation systems [4].

Recently, reinforcement learning (RL) has been an effective method for solving decision-making problems because it can find optimal solutions in uncertain environments and does not require large labeled datasets. However, the dimensionality of the state and action space in mixed autonomy traffic is high. Therefore, applying the RL-based methods usually face the problem of dimensional catastrophe, which significantly reduces efficiency. To extend the RL-based methods to the high-dimensional state and action spaces, deep reinforcement learning (DRL)-based methods have been developed by embedding neural networks into the RL-based methods. In this way, problems in complex and dynamic driving environments with high computational efficiency can be effectively handled without relying on prior knowledge. Therefore, the DRL-based methods have been widely applied to the decision-making process in mixed autonomy traffic [5,6,7,8,9].

To further improve the performance of DRL-based methods, the fusion of graph technology, such as graph representation and graph neural network (GNN), with DRL has attracted a lot of attention in recent studies. This type of method can be termed as a graph reinforcement learning (GRL)-based method. In the field of autonomous driving, GRL-based methods are often used for trajectory prediction [10,11,12], vehicle routing [13], traffic signal control [14,15,16], traffic flow prediction [17,18,19], etc.; they have shown significant advantages. Moreover, some researchers tried to implement GRL-based methods to solve decision-making problems, and ablation experiments in these studies have demonstrated that the GRL-based methods could achieve better performance compared to the DRL-based methods since the incorporation of graph technology can accurately capture topological relationships and model the mutual effect of vehicles [20,21,22].

In summary, the GRL-based methods have great potential to improve the decision-making performance of CAVs in mixed autonomy traffic. Promoting relevant research in GRL-based methods is important for the development of a decision-making system of CAVs. Meanwhile, it is significant to carry out a systematic review of the GRL-based decision-making to provide fundamental understanding, state-of-the-art works, and research directions for relevant researchers. However, recent reviews have mainly focused on DRL-based methods for decision-making and general DRL-based applications in the field of intelligent transportation systems and autonomous vehicles [23,24,25,26]. Thus, this paper presents a comprehensive review of the GRL-based methods for decision-making to fill an important gap for relevant research in the field of GRL-based decision-making for CAVs, including a generic GRL technical framework, a detailed review of the relevant literature, validation methods, and challenges with future research directions. The targeted readers of this paper are the researchers who want to have a jump start in understanding the fundamental DRL and GRL principles of decision-making in autonomous driving, and also researchers who are interested in the field of learning-based decision-making technology for CAVs. We also believe that this paper will serve as a compact handbook of GRL-based methods in decision-making for more experienced researchers to review the existing literature and future challenges. For easy reference, the main acronyms used in this article are listed in Table 1. The main contributions of this paper can be summarized as follows:

- A systematic review of the GRL-based methods for decision-making is presented based on the technical structure of the proposed GRL framework. Related works are clearly summarized in tables for appropriate comparisons.
- A generic GRL framework for the decision-making technology of CAVs in mixed autonomy traffic is proposed. The corresponding elements and functions in the framework are explained in detail.
- Validation methods including evaluation metrics and simulation tools that can be used for the decision-making technology in autonomous vehicles are discussed and summarized for the validation of future related research.
- Challenges and future research topics of the GRL-based methods for decision-making of CAVs are discussed based on the current research status.

**Table 1:** Main acronyms used in this article.

Acronyms	Description
CAV	Connected and Automated Vehicle
HV	Human Vehicle
RL	Reinforcement Learning
DRL	Deep Reinforcement Learning
GRL	Graph Reinforcement Learning
GNN	Graph Neural Network
GCN	Graph Convolutional Network
GAT	Graph Attention Network
ST-GCNN	Spatial–Temporal Graph Convolutional Nerual Network
LSTM	Long Short-Term Memory
GRU	Gate Recurrent Unit
TCN	Temporal Convolutional Network
MDP	Markov Decision Process
POMDP	Partially Observable Markov Decision Process
DQN	Deep Q-Network
D3QN	Double Dueling DQN
PER	Prioritized Experience Replay
AC	Actor Critic
A2C	Advantage Actor Critic
NAF	Normalized Advantage Function
DDPG	Deep Deterministic Policy Gradients
TD3	Twin Delayed Deep Deterministic Policy Gradients
PPO	Proximal Policy Optimization
SAC	Soft Actor Critic

**Figure 1:**
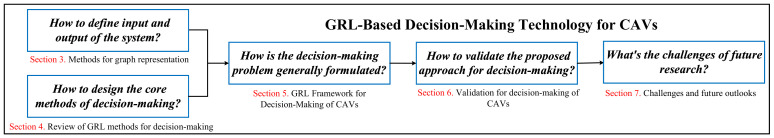
The structure of this paper.

The structure of this paper is illustrated in Figure 1. The rest of this paper is organized as follows. Section 2 summarizes related works and compares them systematically. Section 3 proposes the detailed research methods of this article. Section 4 summarizes the principles and related works of driving scenario construction and graph representation. Section 5 presents a comprehensive review of GRL methods for the decision-making of CAVs, including a summary of typical GNN and DRL algorithms, and overviews of state-of-the-art research. Section 6 introduces a GRL framework for the decision-making of CAVs in mixed autonomy traffic and elaborates in detail on the basic principle, the functionality of the framework, and data flow between different modules. Section 7 proposes the validation approaches for GRL-based decision-making of CAVs. Section 8 prospects the challenges and research topics in future study. Finally, the main conclusions are drawn in Section 9.

## 2. Related Works

Recently, several works have been carried out to summarize the research on decision-making systems in the field of autonomous driving. A summary of related work is shown in Table 2.

In [27], rule-based and deep-learning-based decision-making methods for autonomous vehicles were mainly reviewed; moreover, applications in some existing autonomous vehicles were also summarized. However, RL-based methods were not mentioned in this article. In [28], the combination technology of perception, planning, and decision-making for autonomous vehicles was overviewed. Although this article covered a wide range of categories of decision-making approaches, RL-based methods were still not focused on. In [23], a general framework for decision-making systems of autonomous vehicles was proposed; several categories of methods, including rule-based, deep-learning-based, RL-based, and DRL-based algorithms, were all reviewed. However, the amount of literature summarizing each type of method was inadequate.

Although the above-mentioned literature can help researchers to get a general understanding of decision-making systems for autonomous vehicles, there is still little discussion on DRL-based and GRL-based decision-making methods. To fill these gaps, a survey of DRL-based methods in the field of intelligent transportation systems is provided in [24]. The principle and theory of DRL were summarized, and DRL-based applications for traffic signal control were mainly reviewed. Moreover, in [25], a survey of DRL-based methods for autonomous vehicles was presented. Specifically, a comprehensive review of the basic elements of DRL in each research area of autonomous vehicles (state space, action space, reward functions, etc.) was proposed. Nevertheless, the decision-making approach and GRL-based methods were not discussed in detail in the above two articles. In [26], the typical GRL-based algorithm and application in several fields were systematically overviewed. The GRL-based methods for transportation systems were summarized; however, there was no discussion on decision-making for CAVs using GRL-based methods in this article. In [29], the fundamental knowledge and general technology roadmap from several aspects (environmental perception, decision-making, collaboration, etc.) of CAVs was mainly reviewed. However, the summary of decision-making algorithms was insufficient.

In summary, it is essential to carry out research dedicated to the GRL-based methods for decision-making systems, which we believe is a very timely topic in the field of autonomous driving. Thus, this paper will fill an important gap for relevant researchers interested in GRL-based decision-making for CAVs.

## 3. Research Methods

**Figure 2:**
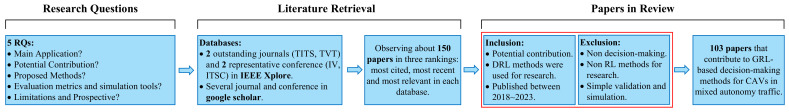
Flowchart of the research methods in this systematic review.

Defining the research method is an important foundation for systematic review work. This section elaborates on the detailed research method of this paper, including three parts: research questions, literature retrieval, and papers in review. The schematic of the proposed research methods is shown in Figure 2.

### 3.1. Research Questions

The proposed research method aims to investigate studies that can contribute to the GRL-based decision-making methods for CAVs. In this paper, five research questions (RQs) were determined for article analysis:

- RQ1: What is the main application of the article?
- RQ2: Which GRL research point could this article potentially contribute to?
- RQ3: What methods does this article suggest around the above research point?
- RQ4: What are the evaluation metrics and simulation methods used by the article to validate the proposed methods?
- RQ5: What are the limitations of the article and the perspective of future research?

### 3.2. Literature Retrieval

The IEEE Xplore and Google Scholar are chosen as the databases to search for articles. In IEEE Xplore, the most authoritative journals and conferences in the field of intelligent vehicles and intelligent transportation systems were selected as the primary sources, which are shown as follows: (1) IEEE Transactions on Intelligent Transportation Systems (TITS); (2) IEEE Transactions on Vehicular Technology (TVT); (3) IEEE International Conference on Intelligent Transportation Systems (IEEE-ITSC); (4) IEEE Intelligent Vehicles Symposium (IEEE-IV). Moreover, to ensure the comprehensiveness of the review, we also conducted overall research in several journals and conferences on Google Scholar.

Then, “*graph reinforcement learning*”, “*deep reinforcement learning*”, and “*decision-making*” were chosen as the main keywords to do the research work. For each journal and conference, articles were saved in three rankings: most cited, most recent, and most relevant articles in each search database. Finally, about 150 articles were selected as potential articles to be cited in this review.

### 3.3. Papers in Review

After identifying the search results, inclusion and exclusion principles need to be clarified for further selection of suitable articles.

The inclusion principles were defined as follows:

- Articles that can potentially contribute to GRL decision-making for CAVs in mixed autonomy traffic.
- Articles that have applied reinforcement learning methods.
- Articles that were published in 2018–2023.

Moreover, the exclusion principles were described as follows:

- Articles that had no relevance related to decision-making technology in any way.
- Articles that did not utilize RL-based methods.
- Articles that had simple and inadequate simulation and validation processes.

## 4. Methods for Graph Representation

For the GRL-based decision-making methods of CAVs, one important issue is to model the driving scenario as a graph and generate graphic environment features. This section explains the basic principle of graph representation of a mixed traffic environment and summarizes related works on different construction methods of graph representation.

### 4.1. Basic Principle

The mixed autonomy traffic is modeled as a graph, where a vehicle is regarded as a node of the graph, and the mutual effect of vehicles is regarded as edges of the graph. The graph is defined as G={N,E}, where $N=\{n_{i},i\in \left\{1,2,\ldots ,n\right\}\}$ is a set of node attributes and $E=\{e_{ij},i,j\in \left\{1,2,\ldots ,n\right\}\}$ is a set of edge attributes; *n* denotes the number of nodes in the constructed graph, and it is equal to the total number of vehicles. In general, the graph representation of the traffic environment consists of the node feature matrix $N_{t}$ and the adjacency matrix $A_{t}$, which are explained in the following.

#### 4.1.1. Node Feature Matrix

The state of vehicles in a mixed traffic scenario is represented by the node feature matrix, whose elements are feature vectors of vehicles. The node feature matrix can be expressed as follows:

(1) $$N_{t}={\left[V_{t}^{1},V_{t}^{2},\cdots ,V_{t}^{i},\cdots ,V_{t}^{n}\right]}^{T}$$

where $\left\{V_{t}^{i},i\in \left[1,n\right]\right\}$ denotes the feature vector of the *i*th vehicle. Feature vectors can contain multi-dimensional data on a vehicle, such as position, speed, and attitude.

#### 4.1.2. Adjacency Matrix

The mutual effect and interaction between vehicles are represented by the adjacency matrix, which can be represented as follows:

(2) $$A_{t}=\left[\begin{array}{cccccc} e_{11} & e_{12} & \cdots & \; & \cdots & e_{1n}\\ e_{21} & e_{22} & \cdots & \; & \cdots & e_{2n}\\ \vdots & \vdots & \ddots & \; & & \vdots \\ \; & \; & & e_{ij} & \; & \\ \vdots & \vdots & \; & & \ddots & \vdots \\ e_{n1} & e_{n2} & \cdots & \; & \cdots & e_{nn} \end{array}\right]$$

where $\left\{e_{ij},i,j\in \left[1,n\right]\right\}$ denotes the edge value of the *i*th and *j*th vehicles; the edge value can be derived through the predefined interaction model of vehicles.

#### 4.1.3. Scenario Classification

Moreover, a certain adjustment to the graph representation is required according to the types of the constructing traffic scenarios. The mixed autonomy traffic scenarios can be divided into open- and closed-loop traffic scenarios according to the invariance of the number of vehicles in a traffic scenario. In an open-loop traffic scenario (e.g., roundabout or ramping scenario), the number of vehicles changes, while in a close-loop traffic scenario (e.g., ring network or vehicle platoon), the number of vehicles is fixed.

It should be noted that for an open-loop traffic scenario, the above node feature matrix and adjacency matrix cannot be directly input into the GRL-based model to generate driving policy. Namely, since vehicles are entering and exiting a given scenario, the number of observed vehicles in the considered road network area changes dynamically. However, in the graph representation process, the features of each vehicle in the considered environment need to be stored in the corresponding position in the feature matrix. In addition, in the action output process, actions executed by a vehicle are defined by elements in the corresponding position in the action matrix. Therefore, an index matrix is required to record the vehicles that currently exist in the open-loop scenario at each time step. Each vehicle is numbered and then recorded in the corresponding location of the index matrix. The index matrix is described as follows:

(3) $$I_{t}=\left[Veh_{1},Veh_{2},\cdots ,Veh_{i},\cdots ,Veh_{n}\right]$$

where $\left\{Veh_{i},i\in \left[1,n\right]\right\}$ indicates the existence of each vehicle; if $Veh_{i}=1$, the ith vehicle exists in the current environment, otherwise $Veh_{i}=0$.

In a closed-loop traffic scenario, features of a vehicle are automatically assigned to a specific position in the feature matrix, and the actions executed by the vehicle are selected from the elements in the corresponding position in the action matrix. Thus, the node feature matrix and the adjacency matrix can be directly input into the GRL module.

In conclusion, we can define ⊙ as the matrix operation at the corresponding position according to the index matrix. Then, the graph representation at the current time step in an open-loop traffic scenario can be formulated as $S_{t}=\left[N_{t},A_{t}\right]⊙I_{t}$; meanwhile, in a close-loop traffic scenario, the graph representation can be directly formulated as $S_{t}=\left[N_{t},A_{t}\right]$.

#### 4.1.4. Scenario Construction

Finally, how to construct an appropriate traffic scenario is the foundation of algorithm simulation and validation. As mentioned before, the mixed autonomy traffic considered in this paper consists of CAVs and HVs, where CAVs are controlled by a GRL-based algorithm. The mixed autonomy traffic model can be constructed according to specific practical conditions.

Decision-making is required in almost all types of driving scenarios, as long as CAVs are in operation status. With the increasing requirements for decision-making systems due to the complexity of the driving environment, related research papers are focusing on V2X cooperation in some typical scenarios, such as general road sections, expressways, urban intersections, merging traffic, and roundabouts [23]. The design of mixed autonomy traffic can refer to the scenarios constructed in some traffic simulation software. Highway-env [30] provides many typical traffic scenarios, such as highways, intersections, and roundabouts. Flow [31] is a DRL-based framework for mixed autonomy traffic, which acts as an interface between traffic simulators (e.g., Sumo [32] and Aimsun [33]) and RL libraries. The Flow framework not only provides typical traffic scenarios, but also creates several benchmarks for the development and verification of RL algorithms; it also supports the import operation of road network files (e.g., OpenStreetMap) to simulate traffic operations under real-world conditions.

### 4.2. Methods for Node Feature Matrix

**Figure 3:**
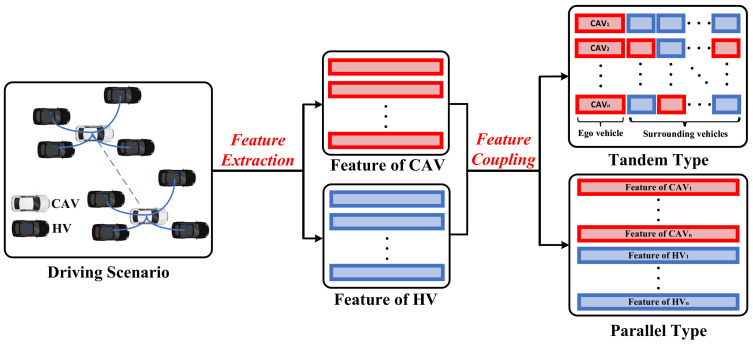
The different construction formulations of the node feature matrix.

This section summarises the methods that can help in constructing the node feature matrix in GRL-based decision-making systems. The key to constructing the node feature matrix is how to couple the vehicle’s state information into feature vectors in accordance with the various driving tasks. During the operation of CAVs, besides the state information of the ego-vehicle, the status of surrounding vehicles also needs to be considered to generate more cooperative driving instructions; therefore, the construction of the node feature matrix requires coupling the information of CAVs with surrounding vehicles. The methods of constructing the node feature matrix can be classified into the tandem type and parallel type according to the coupling mode of information. The construction formulation of the node feature matrix is intuitively described in Figure 3. Various methods for constructing the node feature matrix are presented in Table 3.

#### 4.2.1. Tandem Type

In this paper, tandem type is defined as concatenating the features of CAVs and surrounding vehicle features into the same feature vector. This type of approach only considers other vehicle information within the observed range of the CAVs and is suitable for local decision-making for a small range of CAVs.

In [5], the decision-making problem at a two-lane highway was solved. The features of the ego-vehicle and its four neighboring vehicles were taken into account. The current lane and longitudinal speed of ego-vehicles, and the longitudinal speed and position of neighboring vehicles, were used for state space.

In addition to absolute motion information, relative information of surrounding vehicles can also be considered to construct the state representation. In [34], the multi-vehicle decision-making problem at highway ramps was solved. The relative longitudinal position, speed, and relative lateral position, speed of observed vehicles were selected to construct the state space. Similar research was carried out in [35], the difference was that it solved the decision-making problem of highway lane changing. Moreover, in [36], multi-vehicle decision-making in various driving scenarios was discussed. The speed and position of the ego-vehicle, and the relative speed and position with its following and leading vehicles were all selected to form the state space.

More types of information were considered in [37]. The decision-making problem on a two-lane highway was considered. The features of the ego-vehicle and its nearest front vehicle were coupled. The current lane, current and expected longitudinal speed of the ego-vehicle, the longitudinal speed, and the relative distance of the nearest front vehicle were utilized for state representation. While in [38], information from V2X infrastructure was further considered. Apart from the motion information of the ego-vehicle and the forward vehicle in the current lane, the traffic light information and distance warning messages from the on-board sensor were also utilized. Moreover, in [4], the interaction information was implemented into the feature vector. The surrounding vehicles were classified into different cooperation levels, which were utilized in the state representation to generate more cooperative behaviors for ego-vehicle.

#### 4.2.2. Parallel Type

In this paper, parallel type is defined as storing the features of different CAVs and HVs in the environment into separate feature vectors. This type of approach helps the CAVs to generate globalized driving behaviors, but requires the information of all vehicles within the observation range to be considered to maintain the matrix dimensionality constant, and thus, resulting in increased matrix complexity and higher computational effort.

In [39], the longitudinal position, speed and lateral position, speed of all vehicles in the driving environments were considered to solve the lane-changing problem. In [8], the eco-driving of a vehicle platoon was solved. The features of the whole vehicle platoon were considered. Besides the speed of each CAVs, the relative speed and position of the CAVs within its predecessor and the leading vehicle were also adopted.

Other categories of information can also be coupled in the feature vectors of vehicles. In [14], the traffic signal control problem was solved. For the vehicle node in the state representation, the current speed and lane position of vehicles were considered. In [22,40], the decision-making at highway ramps was solved. All vehicles in the traffic environment were taken into account. The normalized longitudinal speed and position, the current lane, and the driving intention of each vehicle were considered to construct the state representation. Moreover, in [41], the spatio-temporal state construction method was utilized. Specifically, the location information and three-channel records were considered to solve the vehicle dispatching problem.

### 4.3. Methods for the Adjacency Matrix

This section provides an overview of the methods that can contribute to the establishment of an adjacency matrix in GRL-based decision-making systems. The construction of adjacency matrices requires modeling the interactions between vehicles, after which the mutual effects between vehicles are numerically represented and stored in the corresponding positions in the adjacency matrix. Various methods for constructing the adjacency matrix are given in Table 4.

The information sharing between vehicles can be used to construct the adjacency matrix. In [22], the interaction between vehicles was modeled by the connection of different vehicles. The authors assumed that all CAVs were communicable with each other, and CAVs could communicate with HVs within the sensing range. If the two vehicles were connected, the edge value was set to 1; otherwise, the edge value was 0. Moreover, a similar adjacency matrix construction method was carried out in [40]. In [42], the edges in the graph model representing the potential collision relationship between the ego-vehicle and the surrounding vehicles.

The motion information of vehicles is another possible choice to model the interaction between vehicles. In [39], the relative distance between vehicles was calculated to establish the adjacency matrix. Conversely, in [36], a Gaussian speed field based on Gaussian process regression model was proposed. The Gaussian speed field was then fused with the relative speed matrix to construct the adjacency matrix. The proposed method allowed for capturing the spatial and temporal interactions among surrounding vehicles. In [43], a more complete motion representation method was designed. Three directed graph topologies were proposed (view graph, direction graph, and rate graph) to efficiently characterize the asymmetric influence between agents. The relative direction and relative speed of agents were utilized to construct the adjacency matrix.

Other categories of information can also be captured to construct the adjacency matrix. In [44], a bus-pooling system was designed. The adjacency matrix was derived according to the index of vehicles. Specifically, the first row of the adjacency matrix is the one-hot representation of the vehicle i’s index, and the rest are the one-hot representation of its one-hop neighbors’ indices.

**Table 3:** Summary of the node feature matrix constructing methods.

Coupling Type	Refs.	Scenario	Information								
			**Ego-Vehicle**			**Surrounding Vehicles**		**Relative** **Speed**	**Relative** **Position**	**Lane**	**Other**
			**Speed**	**Acceleration**	**Position**	**Speed**	**Position**				
**Tandem**	[5]	Highway cruising	✓			✓	✓			✓	
	[34]	Highway Merging						✓	✓		
	[35]	Lane-changing						✓	✓		
	[36]	Various scenarios	✓		✓			✓	✓		
	[37]	Lane-changing	✓			✓			✓	✓	
	[38]	Highway cruising	✓	✓					✓		Cooperation Level
	[4]	Highway merging	✓	✓	✓						Traffic lights; warning
**Parallel**	[39]	Lane-changing	✓	✓	✓						
	[8]	Vehicle platoon	✓			✓	✓				Vehicle index
	[14]	Traffic signal control	✓							✓	
	[22]	Highway ramping	✓		✓					✓	Driving intention
	[41]	Vehicle dispatching			✓		✓				Channel information

**Table 4:** Summary of the adjacency matrix constructing methods.

Refs.	Scenario	Interaction Model	Model Remarks
[22]	Highway ramping	Information sharing between vehicles.	Interaction between vehicles are defined as 0 and 1 directly based on whether they are connected or not; diverse interaction models need to be considered in the future.
[36]	Various scenarios	Gaussian speed field using the Gaussian process regression (GPR) model.	Both relative distance and relative speed were fused into several kinematic matrices to generate the adjacency matrix.
[42]	Risk Recognition	Potential collision relationship between the ego-vehicle and the surrounding vehicles.	Safety constraints are considered to construct a more complete interaction model to achieve safe and efficient driving.
[43]	Trajectory prediction	Relative direction and relative speed of different vehicles.	Both relative distance and relative speed were taken into account to generate the adjacency matrix.
[44]	Urban bus-pooling	The one-hot representation of the index of the ego-bus and its one-hop neighbors.	The interaction between different vehicles is modeled by a multi-mode adjacency matrix

## 5. Review of GRL Methods for Decision-Making

**Figure 4:**
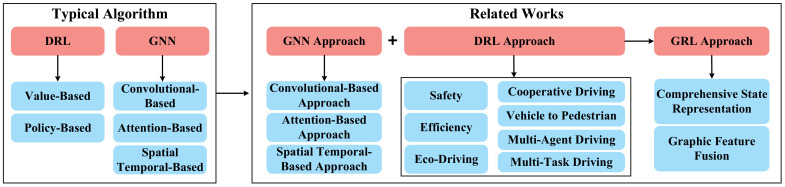
The review structure of GRL-based decision-making methods in Section 5.

This section presents a review of the GRL-based methods for decision-making of CAVs in mixed autonomy traffic. In general, the GRL-based method is a combination of GNN and DRL, the capability of GNN and DRL will both denote a crucial influence on the overall performance of the GRL-based methods. Thus, this section proposes a summary of the following parts: (1) Typical GNN algorithm and GNN approaches that can contribute to the development of GRL-based methods for decision-making; (2) Typical DRL algorithms and DRL-based methods for decision-making; (3) Some existing GRL-based approaches for decision-making. The structure of the proposed review of this section is shown in Figure 4.

### 5.1. Typical GNN Algorithms

This section summarizes some typical GNN algorithms that can be used in GRL-based decision-making to provide readers with an initial understanding of GNN. We first provide some studies that can help to make a start of the GNN method. The original concept of GNN was proposed in [45]. The overviews of various GNN methods and applications were presented in [46,47]. A comprehensive survey of GNN was provided in [48], as well as the open-source scripts, benchmark datasets, and model evaluation of GNNs.

Choosing a suitable GNN method for processing graphic features of the driving environment is crucial to improve decision-making performance. In general, GNN methods applicable to GRL-based decision-making can be divided into convolutional-based methods, attention-based methods, and spatial–temporal-based methods. Convolutional-based methods employ convolution operations on graph-structured data to generate a Euclidean representation of each node in the graph. Attention-based methods introduce attention mechanisms to assign different weights to different nodes in a neighborhood, allowing to learn the relative weights between two connected nodes and reducing local manipulation of graphs to improve computational efficiency. Spatial–temporal-based methods adopt temporal neural networks (e.g., LSTM and TCN) to process time sequential sets of graphic features over a sequence of time, which helps to generate driving instructions with high priority. Several typical GNN algorithms are presented in Table 5.

### 5.2. Review for GNN Methods

Although there has been little research into the direct application of GNN methods to the decision-making of CAVs, GNNs have a wide range of other applications in the field of intelligent transportation systems, such as traffic signal control and traffic flow prediction. The GNN methods used in these works can effectively encode the traffic environment, and there is great potential to improve performance by migrating these GNN methods to GRL-based decision-making systems. Thus, this section reviews relevant GNN approaches which can be used in the development of GRL-based methods for decision-making. The summary of the discussed GNN approaches is shown in Table 6.

#### 5.2.1. Convolutional-Based Methods

In [59], GraphSAGE was utilized to achieve traffic flow forecasting. A case study was proposed in the urban area of Hangzhou, China; results showed that the proposed method performed well in short-term prediction. In [43], the trajectory prediction issue was solved. Three directed graph topologies were proposed (view graph, direction graph, and rate graph). Typical GCN was utilized to process the fused graphic features. The method was validated on four scenarios in the Stanford Drone Dataset.

#### 5.2.2. Attention-Based Methods

In [60], a GRL-based approach was carried out to solve the network slicing management. Specifically, GAT was implemented into typical DQN and A2C frameworks. Results showed that the GAT-DQN performed the best among the baselines and all designed methods. In [61], a GRL-based approach was proposed for traffic signal control. An Actor-Critic framework was utilized, and a GAT model was implemented into the critic network to learn the spatial feature of the surrounding intersection. Results demonstrated that the proposed method outperformed the traditional and state-of-the-art DRL-based control methods. An urban bus-pooling system was designed in [44]. A double graph attention actor-critic (DGAAC) framework was established by integrating high-level and low-level actor-critic frameworks with GAT. The experiment was carried out based on real-world datasets in Shenzhen, China. Results showed that the proposed methods could outperform all baselines.

#### 5.2.3. Spatial–Temporal-Based Methods

A straightforward approach is to combine a typical GCN with a temporal neural network to handle the spatial–temporal features. GRU has been widely selected to combine with GNN. Because the GRU model has simple structure and is faster to train, making it is suitable for building larger networks and solving complicated tasks. In [62], a Temporal Multi-Graph Convolutional Network (T-MGCN) was proposed for traffic flow forecasting, consisting of a combination of multi-layer GCN and GRU. In [63], a knowledge-driven spatial–temporal graph convolutional network (KST-GCN) was proposed for traffic forecasting. The GCN and GRU were fused, and a Knowledge Fusion Cell (KF-Cell) was further designed to combine the knowledge and traffic features as the input of the proposed network. Similar work was carried out in [64], where the proposed method was evaluated on three real-world case studies, and the experimental results show that the proposed method outperforms state-of-the-art traffic prediction methods. The evaluation was carried out on two real-world traffic datasets and observed improvement by approximately 3% to 6% as compared to the state-of-the-art baseline.

Other types of temporal neural networks can also be implemented into the GNN framework. In [19], a hierarchical traffic flow forecasting network was proposed by fusing the GCN and LSTM. Specifically, an attention fusion mechanism is further designed to combine the long term with the short term in order to mitigate the over-smoothing problem of GCN. Results showed that the proposed method was effective in three public traffic flow datasets. In [65], a Hybrid Spatio–Temporal Graph Convolution Network (HSTGCN) was proposed by aggregating TCN and GCN to predict the future traffic speed. The overall mean absolute percentage error of the proposed method was between 9 and 13%. In [66], a spatial–temporal graph-based transformer model (STGT) was proposed by combing GCN and a transformer model. Specifically, GCN was used to extract the spatial information, and the transformer model exploited the temporal information. Results showed better performance on PeMSD8 datasets.

To represent the mutual effects between different nodes of the constructed graph in a more comprehensive way, GAT has become another possible choice for integration with temporal neural networks. In [67], a traffic-gated graph neural network (Traffic-GGNN) was proposed to solve the traffic flow forecasting problem. Specifically, the GRU was combined with self-attention GNN. Results yield better efficiency and effectiveness on three real-world datasets (SZ-taxi, Los-loop, and PEMS-BAY). In [17], a spatial–temporal Attention Neural Network (STAtt) was proposed to solve the traffic flow forecasting. The GAT and LSTM algorithms were combined to describe the variability of the roads’ interactions. Results showed that the proposed model can achieve good performance in the short-time prediction task within 1 h. A similar method named EA-Net was proposed in [10] to solve the trajectory prediction problem by combining the GAT and LSTM. The method was verified on NGSIM and highD dataset. The results showed that the prediction accuracy of the proposed Environment-Attention Network in the two datasets is more than 20% higher than that of the single-structure model.

In [68], trajectory prediction was solved by GNN. A spatio–temporal graph dual-attention network was proposed to process the history data, as well as a topological attention layer that updated node attributes from the spatial or topological perspective, and a temporal attention layer that outputs a high-level feature embedding for each node. The experimental results demonstrated that the model achieved better performance than various baseline approaches in terms of prediction and tracking accuracy.

**Table 6:** Summary of the related GNN approaches.

Category	Refs.	Scenario	Models	Basic Modules	Simulator/Dataset
**Convoluational-** **Based**	[43]	Trajectory prediction	-	GCN, three directed graph	Stanford Drone Dataset.
	[59]	Traffic speed forecasting	-	GraphSAGE	Urban area in Hangzhou, China.
**Attention-** **Based**	[44]	Bus-pooling	DGACC	GAT, Hierarchical AC	Real-world datasets in Shenzhen, China.
	[61]	Traffic signal control	-	GAT, AC	Real-world datasets from New York, Hangzhou, and Jinan.
	[60]	Slicing resource management	-	GAT+ DQN, GAT+A2C	Numerical analysis.
**Spatial–Temporal-** **Based**	[62]	Traffic flow forecasting	T-MGCN	Multi-layer GCN, GRU	HZJTD, PEMSD10.
	[63]	Traffic flow forecasting	KST-GCN	GCN, GRU	Dataset from Luohu District, Shenzhen, China.
	[64]	Traffic speed/flow forecasting	OGCRNN	GCN, GRU	D.C., Philadelphia, and PeMSD4.
	[19]	Traffic flow forecasting	LTT+STGC	GCN, LSTM	PeMSD7(M), PEMS-BAY, and Beijing Metro datasets.
	[65]	Traffic flow forecasting	HSTGCN	GCN, TCN	Traffic data from the Shenzhen Urban Traffic Planning Centre.
	[66]	Traffic flow forecasting	STGT	GCN, transformer model	PemsD8.
	[67]	Traffic flow forecasting	Traff-GGNN	Self-attention GNN, GRU	SZ-taxi, Los-loop, and PEMS-BAY Dataset.
	[17]	Traffic flow forecasting	STAtt	GAT, LSTM	Road section in Beijing, China.
	[10]	Trajectory prediction	EA-Net	GAT, LSTM	NGSIM, highD.
	[68]	Trajectory prediction	STG-DAT	Attention mechanism, GRU	ETH, UCY, SDD, ID, and Standford Drone Dataset.

^1^ It should be noted that the models proposed in the cited research papers are composed of several basic models; thus, for the strengths and weaknesses of these models, please refer to Table 5.

### 5.3. Typical DRL Algorithms

A preliminary understanding of typical DRL algorithms is the basis for subsequent decision-making research. Here, we summarize some studies that can help to achieve a basic understanding of DRL algorithms. The fundamentals and future developments of existing DRL algorithms were proposed in [69]. An overview of fundamentals, principles of typical algorithms, applications, and open resources of DRL was provided in [70]. A survey of the implementation principle of DRL for autonomous driving can refer to [24,25]. Moreover, this section also discusses the characteristics of some typical DRL algorithms. Several typical DRL algorithms are presented in Table 7.

### 5.4. DRL-Based Methods for Decision-Making

The DRL module is an important part of the GRL-based decision-making systems and has a significant impact on the performance of decision-making. Numerous studies have focused on DRL-based methods to solve the decision-making problem in mixed autonomy traffic. These studies that focus on DRL-based decision-making can make a significant contribution to GRL-based methods. Thus, this section proposes a review of state-of-the-art literature on DRL-based decision-making methods.

Moreover, it is crucial to categorize the relevant literature in a proper way, considering that the purpose of DRL-based works is to deal with the current research problem of autonomous driving. For this reason, we have identified topical issues based on current research and developed the following review of these issues. Several exemplary works are summarized in Table 8.

#### 5.4.1. Safety

Safety is the first priority in cooperative decision-making. Learning how to drive safely is essential for CAVs in mixed autonomy traffic.

A primary possible solution for designing a safe policy to define additional restrictions on action selection. In [85], the DQN was combined with formal safety verification to ensure that only safe actions could be selected, and highly desired velocity was reached with nearly no collision. However, the trade-off between safety and efficiency should be further considered. In [86], a risk-sensitive approach was proposed in the T-intersection scenario; offline distributional DQN was used to solve the model, and an online risk assessment was performed to evaluate the probability distribution of the generated actions. The results showed that the collision rate was less than 3%. Similarly, in [87], a “model-checker”-based safety RL method was proposed to guarantee the safety of intersections in complex environments. A recurrent neural network was trained to generate beliefs, and driving instructions were generated based on the DQN and according to the constraints of the safety threshold. Approximately 100 steps were necessary to complete the goal for the given scenario at a low collision rate. In [88], a safe decision-tree policy was designed to ensure safe distance in a highway overtaking scenario; collision was obviously reduced in randomized initialization. However, the reward function needed further development because the overall reward decreased when collisions were reduced.

Another possible solution for ensuring driving safety is constructing a safe reward function to train a DRL-based model to generate safe driving behaviors. In [89], a strict risk-based reward function was derived to punish risk situations instead of only collision-related ones. A generic “risk-aware DQN” was proposed for generating safe behaviors. Results showed that a success rate of near 95% could be achieved under a low training vibration. Moreover, in [90], an assessment module based on the Bayesian inference was designed to ensure safe reward generation. In [91], the trade-off between safety and agility was considered when designing the reward function; overall braking induced by the lane-changing behaviors was mainly minimized while encouraging the AV to speed up.

Several other techniques have also been adopted to improve driving safety. In [92], multiple neural networks were assembled with additional randomized prior functions to optimize the typical DQN capacity. In this way, safe driving could be realized in more uncertain intersections. Results showed that the success rate of more than 95% could be achieved under a collision rate of less than 5%. However, the constructed simulation environment was too simple. In [93], adversarial robust control (ARC) was implemented in a highway leader–follower driving scenario. The A3C was selected as a basic framework, and a protagonist network was constructed to control the follow vehicle, whereas an adversary network was constructed to control the lead vehicle. The number of collisions decreased by 90.25%. Nevertheless, the non-leader–follower scenario should be also considered. In [94], an attention mechanism was introduced to focus on more spatially and temporally important environmental features to generate self-attention features. This enabled safe and efficient driving decisions even under noisy sensory data, and a success rate of more than 87% was achieved at a low collision rate and average braking time.

#### 5.4.2. Efficiency

Another critical research topic in decision-making is how to ensure high efficiency. In this study, efficiency refers to solve the DRL model of decision-making with high real-time performance, which is critical to practical applications in CAVs.

In [95], the rainbow DQN was combined with a safely driving rewarding scheme to achieve high sample efficiency. The trained model converged to stable reward after only 200k training steps compared with baseline (1M training steps). In [96], decision-making at an intersection was modeled as hierarchical-option MDP (HOMDP), where only the current observation was considered instead of the observation sequence over a time interval to reduce the computational cost. A success rate of more than 97% was achieved, and 50% fewer number of steps were needed to finish the driving task compared with the baseline. In [97], human demonstration with the supervised loss was implemented into the training of a double DQN for a better exploration strategy to boost the learning process. A success rate of over 90% could be reached with only 100 training epochs.

#### 5.4.3. Eco-Driving

Eco-driving can reduce resource waste and have significant economic benefits. Learning how to control the ego-vehicle more efficiently and cooperate with other vehicles to improve transportation efficiency could be beneficial to energy saving.

Vehicle platoon control has been a hot topic because improper driving behavior of a vehicle can adversely affect the driving efficiency of other vehicles. In [98], a hybrid DRL and genetic algorithm for smart-platooning (DRG-SP) was proposed. A genetic algorithm was implemented into the DRL-based framework to overcome the slow convergence problem and ensure long-term performance. The driving policy was updated through a rank-based replay memory to make highly optimal decisions. Results showed that the energy consumption was reduced by 8.57% while maintaining high efficiency. In [8], a communication proximal policy optimization (CommPPO) was proposed for eco-driving. A predecessor–leader–follower typology in the platoon was utilized with a new reward communication channel to guarantee efficient information transmission and avoid the lazy-agent problem. In addition, curriculum learning was first adopted to train a small-size platoon to facilitate the training process of the whole vehicle platoon. Results showed that fuel consumption was reduced by 11.6%. In [99], a DRL approach was carried out to solve the decision-making of a mixed vehicle platoon. Specifically, augmented random search (ARS) was proposed to deal with the delayed reward. Results showed that when the travel delay is not sacrificed, the proposed control method can save up to 53.64% electric energy.

Several other driving scenarios have also been investigated in the research on eco-driving. In [100], an “I-210 network” was designed. Multi-agent PPO with a traffic smoothing controller was proposed to eliminate traffic shockwaves. The designed system achieved a 25% fuel consumption reduction at a 10% penetration rate. However, only two vehicles were controlled in the constructed scenario. In [101], an efficient on-ramp merging strategy (ORMS) was proposed. The D3QN was combined with prioritized experience replay to learn the lane-changing behaviors, and a motion planning algorithm based on time-energy optimal control was developed by adding time term into the reward function to generate an optimal trajectory. Results showed that the fuel economy and traffic efficiency could be improved by 43.5% and 41.2%. In [38], a unity-based simulator was developed, and a mixed traffic intersection scenario was designed. A hybrid RL (HRL)-based framework, which combined the rule- and DRL-based modules. was proposed for eco-driving at intersections. Particularly, the rule-based module was used to ensure good collaboration between the two types of strategies, while a dueling DQN was implemented into the DRL module to generate driving behaviors by capturing both visual and logical information. Results showed that energy consumption and travel time were reduced by 12.70% and 11.75%, respectively.

#### 5.4.4. Cooperative Driving

In this study, cooperative driving mainly refers to the decision-making of a single CAV considering collaboration with other HVs. Learning how to perform cooperative driving behavior in mixed autonomy traffic has significant implications for improving traffic efficiency.

The highly comprehensive modeling of interactions has great potential to improve cooperation between vehicles. In [4], HVs were modeled with different cooperation levels in the DRL framework. Typical DQN was combined with a belief updater to generate driving instructions under different cooperation levels. The number of time-out failures was obviously reduced compared with the baseline. Moreover, in [102], a multi-agent RL method for harmonious lane-changing was developed. The proposed harmonious driving method relied only on the ego-vehicles’ limited sensing results to balance the overall and individual efficiencies. In addition, a reward function that combined individual efficiency with the overall efficiency for harmony was designed. Results showed that a high mean vehicle flow rate could be reached under congested conditions.

Better prediction of other vehicles’ behaviors can help to generate cooperative behaviors of the ego-vehicle. In [103], the Deep-Sets DQN was proposed to handle the dynamic number of vehicles. The proposed model can efficiently predict cooperative drivers’ behaviors based on their historical data and generate high-level cooperative instructions; the MPC was used to generate driving trajectories. Similarly in [104], a high-accuracy data-driven model was developed based on a directed graphic model to predict the intention of HVs. The predicted results were then input into the DRL framework to generate cooperative driving behaviors. Results showed that an average speed of 31.8 m/s could be reached with stable speed deviation.

Moreover, obtaining information on the local driving environment from roadside infrastructure to make a preliminary assessment of the driving situation can be greatly helpful to achieve cooperative driving of CAVs. In [105], a cooperative decision-making scheme for CAVs in an emergency was proposed. At first, the traffic state was observed from the roadside infrastructure to judge whether an emergency will occur for each CAV. Then, Double DQN was utilized to evaluate all potential emergency destinations for collision avoidance. Finally, a safety evaluation map was established according to the evaluation result of the DRL model to generate driving behaviors for CAVs. Results showed that the driving reward could be increased obviously.

#### 5.4.5. Vehicle-to-Pedestrian Interaction

Apart from the cooperation between vehicles, vehicle–pedestrian interaction is also important for safe autonomous driving.

One solution for ensuring pedestrian safety is to generate vehicle braking commands directly. In [106], an autonomous braking system based on a DQN was designed. The output of the system was a series of braking commands of different strengths. The collision rate reaches zero when the time-to-collision (TTC) interval was longer than 1.5 s. However, only a single vehicle and person were considered in the constructed scenario. In [107], a multi-objective reward function was designed in the DQN framework for navigation in urban environments in the presence of pedestrians. Both acceleration and braking commands were generated, and the results indicate that both driving safety and efficiency were optimized.

Predicting pedestrians’ behaviors could contribute to the safe driving of autonomous vehicles. In [108], a safe speed network was constructed and integrated with the DRL agent. Moreover, a risk assessment was performed to predict the behaviors of distracted pedestrians. The predicted results were then input into the double DQN with an integrated safe speed network to generate driving behaviors.

Complete modeling of pedestrians’ intentions is important since pedestrians’ behaviors are highly uncertain. In [109], a pedestrian was modeled as a DRL agent to define the vehicle–pedestrian interaction as a multi-agent DRL problem. Two levels of the pedestrian models and vehicle models were established. The obtained results indicated that a collision rate of 0.135% was achieved under maximal noisy level, and the DRL pedestrian model could learn an intelligent crossing behavior. However, whether modeling the pedestrian as a DRL agent could reduce the requirement for vehicle intelligence should be further explored.

#### 5.4.6. Multi-Agent Driving

In general, multiple CAVs need to be controlled in mixed autonomy traffic. Thus, multi-agent decision-making technology is highly demanded.

In [110], a simple multi-agent DRL framework was proposed to solve the problem of a highway merging scenario. The acceleration command of each AV was generated using the status of other vehicles as input data. Collision-free performance was achieved at an on-ramp length of 70 m or longer with vehicles that were 5 m or more apart. However, only two vehicles (one driving on the main lane, another driving on the merge lane) were controlled in the constructed scenario; additional vehicles should be considered. More vehicles were considered in [111], where the REINFORCE algorithm was used to generate driving behaviors based on local observations for an arbitrary number of controlled vehicles. Results showed that a near-optimal throughput with 33–50% controlled vehicles could be achieved.

A more complete multi-agent decision-making system was designed in [35], and a multi-agent A2C method with the parameter-sharing mechanism and multi-objective reward function was proposed to achieve decentralized control of multiple AVs. Feature vectors of the ego-vehicle and its neighboring vehicle were used as input data, and driving instructions of all AVs were then generated. Moreover, the designed reward function was used to evaluate the performance of every single AV, and the transition of each vehicle was stored into experience replay individually. Then, the experience replay was sampled for model training. The authors conducted similar research in [34], where the main improvement was that a priority-based safety supervisor was developed to avoid invalid behaviors to reduce collision numbers.

Modeling the interaction between different vehicles can provide more reasonable driving behaviors for each vehicle. In [5], a dynamic coordination graph was proposed to model the continuously changing topology during vehicles’ interactions. Tubular Q-learning was proposed to generate driving behaviors. In addition, two mechanisms (the global coordination mechanism and the local coordination mechanism) were employed to extend the approach to more general and complex situations with any number of vehicles. Results indicated good performance in scenarios with different numbers of vehicles. In [112], an advanced Reinforced Autonomous Intersection Management (adv.RAIM) was proposed to solve multi-agent decision-making at intersections. LSTM cell was implemented for each surrounding vehicle to continuously encode the speed interaction between different vehicles. Results showed that the proposed methods reduced the waiting time by 56% compared with other recently proposed AIMs.

#### 5.4.7. Multi-Task Driving

Driving efficiency of CAVs can be further improved by optimizing multiple driving tasks simultaneously.

A straightforward approach for optimizing multiple driving tasks simultaneously is to establish multi-objective reward functions to train an AV to execute multiple driving tasks simultaneously. In [113], a unified four-dimensional vectorized reward function was derived and combined with a DQN to solve the navigation problem at different types of intersections. The designed reward function consisted of the reward values generated by four different driving actions in the current state. In [114], two objectives, collision avoidance for safety and jerk minimization for passenger comfort, were investigated in designing the reward function. The DDPG was used for behavior generation, and results showed that vehicle jerk is reduced by 73% with nearly no collision in the highway merging scenario. Similarly, in [115], driving speed and fuel efficiency were jointly considered in designing the reward function. The AC algorithm, which takes the visual image as input and outputs the control commands to achieve the end-to-end driving, was used. However, the verification scenario included only the ego-vehicle but no other vehicles.

In [116], more types of objects were implemented into the reward function. Safety, comfort, economy, and transport efficiency were considered in designing a multi-mode reward function. The PPO was employed, and results indicated that a feasible and effective driving policy for autonomous electric vehicles was achieved. However, more combinations of weight coefficients need to be investigated. In addition in [117], similar objects were considered in the design of the reward function. The main difference was that meta RL was adopted to improve the generalization capability of the DRL model for more complex environments. The overall success rate was up to 20% higher than the benchmark model, and the collision rate was reduced by 18%.

Decoupling the driving tasks into several subtasks is another possible solution for dealing with multi-task driving. In [118], the driving tasks were modeled through a hierarchical framework integrating high-level policy and low-level control. High-level driving behaviors were generated by the A2C and then input into the vehicle kinematic model to generate acceleration and steering angle commands. Results showed that the collision rate was less than 5%. In [119], the driving tasks were decomposed into several simple tasks, and a hierarchical program-triggered RL-based (HPRL) framework was established to train different agents to complete the decomposed subtasks simultaneously. The proposed method demonstrated good training efficiency in multi-task autonomous driving.

#### 5.4.8. Other

Some other research objectives have also been considered in recent studies. In [120], driving ethics were considered, including three different policies, particularly, Rawlsian contractarianism, utilitarianism, and egalitarianism. A search-based method was used to generate ethical driving instructions. In [121], the benchmark establishment process was mainly studied, and an OpenDS-CTS benchmark based on the major German in-depth road accident study GIDAS was proposed to verify safe decision-making in vehicle–pedestrian accident scenarios. Moreover, a hybrid method named HyLEAP, which combines a belief tree and DRL, was proposed to generate collision-free behaviors.

### 5.5. Review for GRL Methods

The DRL-based methods are prevalent for decision-making in mixed autonomy traffic. However, when employing only DRL to solve multi-vehicle decision-making and cooperative driving, system complexity increases significantly, and it is difficult to model relationships between vehicles. Since a GNN can obtain the topological relationships and facilitate the modeling of the mutual effects of multiple vehicles, it has great potential to improve decision-making performance in mixed autonomy traffic. For this reason, this section summarizes the existing relative research on the GRL-based methods for decision-making of CAVs. An overview of the GRL-based approaches is given in Table 9.

#### 5.5.1. Comprehensive State Representation

A straightforward solution has been to model the mixed autonomy traffic as a graph, representing features of vehicles as a node feature matrix and mutual effects between vehicles as an adjacency matrix. Therefore, a GNN can be used to aggregate the above two matrices into a DRL-based framework to generate driving behaviors. In [22], a highway ramping scenario was constructed and modeled as an undirected graph. The GCN was used to acquire the data collected through collaborative sensing, while cooperative lane-changing decisions were generated by the DQN. The results showed that the average reward was higher than those obtained by the rule-based and LSTM methods in different traffic densities. However, the generated behaviors did not correspond to the current vehicles. Based on [22], two improvement solutions were proposed. In [122], a generalized single-agent GRL training method was developed. The training results were applied to multi-agent training to reduce the computational cost. However, continuous action space should be considered for generating acceleration commands. In [123], a multi-mode reward function with a decision-weighted coefficient matrix was derived to train multiple decision-making modes in different traffic scenarios. Four decision-making strategies, including aggressive incentive (AGGI), aggressive punishment (AGGP), conservative incentive (CONI), and conservative punishment (CONP), were trained with a multi-step double DQN. Results showed that higher reward and average speed could be achieved.

Exploring additional ways of modeling interactions between vehicles is significant to improving the effectiveness of the GRL-based methods. In [39], a highway lane-changing scenario was modeled as a directed graph, and graph representation was implemented based on the relative position between vehicles. Furthermore, in [124], an intersection scenario was constructed, and the connection between vehicles was modeled based on their turning intentions. In [36], an attention mechanism was introduced to capture the mutual interplay between vehicles to achieve better cooperative control. Moreover, a dynamic adjacency matrix based on the Gaussian speed field using the Gaussian process regression (GPR) model was constructed to capture spatial and temporal interactions between surrounding vehicles. A graph attention network (GAT) was used for graphic feature extraction, while the PPO was employed for policy generation. Various scenarios were verified, and results indicated a higher average reward than that of the baseline.

**Table 8:** Summary of exemplary DRL-based approaches for decision-making in mixed autonomy traffic.

Task Solved	Refs.	Methods	Scenario	Verification	Performance	Characteristics	
						**Main Solution**	**Remarks**
Safety	[86]	Distribu- tional DQN	Intersection	Numerical simulation	Collision rate of less than 3%.	Safe policy	An online risk assessment mechanism is introduced to evaluate the probability distribution of different actions.
	[89]	Risk-aware DQN	Intersection	Simulation in Carla	More than 95% success rate with steady performance.	Safe reward function	A stricter risk-based reward function is constructed to solve the model.
	[93]	SAC	Various scenarios	Simulation in Carla	Success rate of more than 87% with a low collision rate.	Attention mechanism	An attention-based spatial–temporal fusion driving policy is proposed.
High efficiency solving	[96]	DQN	Intersection	Simulation in SUMO	Over 97% success rate with a small total number of finishing steps.	Hierarchical framework	Hierarchical Options MDP (HOMDP) is utilized to model the scenario.
	[97]	Double DQN	Highway lane-changing	Numerical simulation	Over 90% success rate is achieved with only 100 training epochs.	Demonstration	Human demonstration with supervised loss is introduced.
Eco- driving	[8]	PPO	Vehicle platoon	Simulation in SUMO	Fuel consumption is reduced by 11.6%.	Oscillation resuction	A predecessor–leader–follower typology is proposed.
	[38]	Dueling DQN	Intersection	Unity Engine	Energy consumption is reduced by 12.70%.	Hybrid framework	The rule-based strategy and the DRL strategy are combined.
Coopera- tive driving	[102]	DQN	Highway lane-changing	Numerical simulation	Mean vehicle flow rate of 6529 in congested conditions.	Behavior prediction	Individual efficiency with overall efficiency for harmony is combined.
	[103]	Deep-Sets DQN	Highway merging	Numerical simulation	Low comfort cost is achieved under cooperative driving.	Behavior prediction	Cooperative drivers are identified from their vehicle state history.
Vehicle to Pedestrian	[106]	DQN	Pedestrian crossing	Simulation in PreScan	Collision rate reaches zero when TTC is higher than 1.5 s.	Brake Control	An autonomous braking system is designed with different braking strengths.
	[108]	Double DQN	Distracted pedestrian crossing	Simulation in OpenDS	Different safe speed ranges are verified under various pedestrian situations.	Behavior prediction	A risk assessment is performed to predict the behaviors of pedestrians.
Multi-agent driving	[110]	DDPG	Highway merging	Numerical simulation	Collision-free performance is achieved at the merging ramp.	Parameter sharing	Collision avoidance is emphasized in the interaction between vehicles.
	[34]	Improved A2C	Highway merging	Simulation in Highway-env	Zero collision rate is achieved in three tested modes.	Parameter sharing	A priority-based safety supervisor is developed to reduce collision.
	[5]	Tubular Q-learning	Highway cruising	Graphical simulation	High average reward with good lane-keeping behaviors.	Interaction modeling	A dynamic coordination graph is proposed to model the interactive topology.
Multi-task driving	[113]	Multi- task DQN	Intersection	Simulation in SUMO	Success rate is higher than 87%.	multi-objective reward function	Multiple tasks are represented by a unified four-dimensional vector with a vectorized reward function.
	[114]	DDPG	Highway merging	Simulation in SUMO	Vehicle jerk is reduced by 73% with nearly no collision.	multi-objective reward function	Collision avoidance for safety and jerk minimization for passenger comfort are both investigated.
	[119]	DQN\DDPG	Various scenarios	Simulation in Carla	100% success rate with no traffic rule violations.	Tasks decoupling	Multiple agents are trained with different simple tasks under the hierarchical DRL framework.

#### 5.5.2. Graphic Feature Fusion

Another method is to use a GNN to fuse multiple feature categories without modeling the mixed autonomy traffic as a graph. In [125], various traffic scenarios were designed in the Carla simulator. Graph node features of vehicles and bird-eye view images were concatenated and input in the GAT. Then, the aggregated features were fused with the motion vector and route of the ego-vehicle and fed to a multi-layer perceptron (MLP) model to generate throttle and steering commands. Safe navigation in a complex driving environment was achieved while satisfying traffic rules. Similar research was conducted in [126]. The main difference was that only graph node features and bird-eye view were fused and input in the GAT. The D3QN was combined with a noisy network to improve policy exploration and generation. Results showed that a success rate of over 96% was achieved in training scenarios.

**Table 9:** Summary of the GRL-based approaches for decision-making in mixed autonomy traffic.

Refs.	Methods	Scenario	Verification	Performance	Characteristics	
					**Main Solution**	**Remarks**
[22]	GCN+DQN	Highway ramping	Simulation in SUMO	Better than those of the rule-based and LSTM at different traffic density values.	Graph modeling	The traffic scenario is modeled as an undirected graph. However, the generated behaviors do not correspond to the current vehicles.
[122]	GCN+DQN	Highway ramping	Simulation in SUMO	The network convergence and training efficiency are improved.	Graph modeling	A generalized single-agent GRL training method is proposed and extended to the multi-agent framework.
[123]	GCN+DQN	Highway ramping	Simulation in SUMO	High reward and average speed can be achieved.	Graph modeling	A multi-mode reward function with a decision-weighted coefficient matrix is derived to achieve the training of multiple decision-making modes.
[39]	Directed graph+PPO	Highway lane-changing	Numerical simulation	An 81.6% success rate is achieved at 11.1% collision rate.	Graph modeling	Graph representation is implemented based on the relative position between vehicles.
[124]	GCN+TD3	Intersection	Simulation in Highway-env	Flow rate in the intersection is significantly improved.	Graph modeling	The varying number of vehicles in the scenario is handled by a flexible graph representation.
[36]	GAT+PPO	Various scenarios	Simulation in SUMO	Average reward is increased in all the tested scenarios.	Graph modeling	The attention mechanism is introduced to capture mutual interplay among vehicles to achieve better cooperative control.
[125]	DiGNet	Various scenarios	Simulation in Carla	Safe navigation in a complex driving environment while obeying traffic rules.	Graphical feature fusion	Graph representation is fused with bird’s-eye views of the driving scenario and route information.
[126]	GAT+D3QN	Various scenarios	Simulation in Carla	Over 96% success rate in the training scenarios.	Graphical feature fusion	Graph representation is fused with bird’s-eye views. The PID controller is implemented in the decision-making module.

## 6. GRL Framework for the Decision-Making of CAVs

Before carrying out specific research, it is crucial to get a comprehensive understanding of the technical framework of the GRL-based decision-making system and to break it down into different modules. This can be very helpful for researchers to clarify the function of each module in the framework, and which parts can be used to start with methodological innovations. Therefore, this section proposes a generic GRL-based decision-making technical framework for CAVs based on the previous sections of the literature review to extract research topics and the relationship between different research points. The architecture, basic principle, and important variables are systematically described in the following sections.

### 6.1. GRL Framework Architecture and Principle

**Figure 5:**
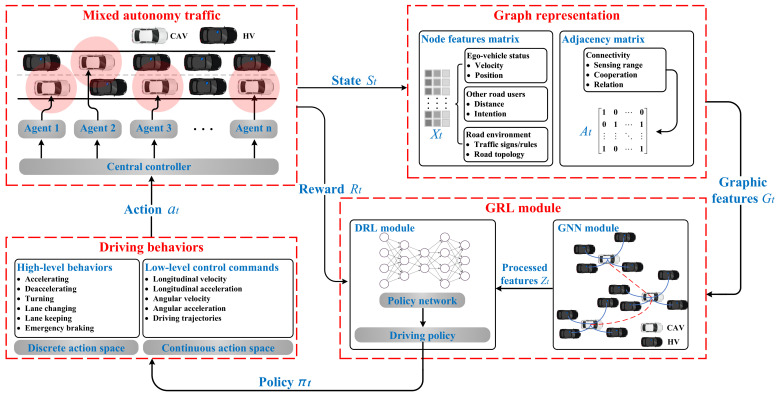
The schematic diagram of the proposed GRL-based decision-making technical framework.

The complete design of the proposed framework is illustrated in Figure 5. The presented GRL framework contains the following modules: mixed autonomy traffic module, graph representation module, GRL module (including GNN and DRL module), and driving behaviors module. The mixed autonomy traffic module is the basis of the proposed framework. The graph representation module is used to generate graphic features of mixed autonomy traffic and input them into the GRL module. The GRL module is the core of the framework to generate driving policies. The driving behavior module selects driving behavior according to the driving policies and inputs it to the mixed autonomy traffic to update the environment state.

The main characteristics of the GRL-based methods can be summarized as follows: (1) Mixed autonomy traffic is modeled as a graph. Particularly, a vehicle is regarded as a node of the graph, while the mutual effects of vehicles are regarded as edges of the graph [21]; (2) A GNN is adopted for feature extraction; extracted features are fed to the policy network to generate the driving behaviors of CAVs.

The general decision-making problem can be modeled as a finite horizon Markov decision process (MDP) [127] or partially observable Markov decision process (POMDP) [128] according to the observability of the environment. For the operation process of CAVs, information can be shared between vehicles through the vehicular network, and vehicles can obtain information about the driving environment through road infrastructure, thus the driving environment can be considered to be fully observable. Furthermore, the GRL-based decision-making problem of CAVs addressed by the constructed framework is a multi-agent decision-making problem that requires consideration of environmental observations from a previous period of time sequence. Therefore, the temporal graphical Markov decision process (TGMDP) is proposed in this paper to model the decision-making problem of CAVs from the temporal and spatial dimensions.

TGMDP is defined by tuple $\left(S^{n,T},A^{n},F,R,\gamma \right)$, where *n* represents the number of controlled CAVs in the driving environment and *T* represents the length of time sequence. It should be noted that if n=1 and T=1 are both satisfied, the TGMDP describes a non-temporal single-agent decision-making process; in this case, TGMDP is the same as a typical MDP. Furthermore, $S^{n,T}$ denotes a set of states that represent the current temporal observation of mixed autonomy traffic. $S^{n,T}$ is the fundamental data input of the decision-making algorithm in the CAV environment, and $S^{n,T}$ is acquired through CAV observations of the simulation environment. $A^{n}$ is a set of actions performed by the multiple CAVs. Specifically, $A^{n}$ is defined according to the driving maneuvers that CAV can adopt in the defined driving environment. *F* represents the transition probability function to describe the probability of the controlled CAVs taking an action set $a_{t}^{n}$ at a certain time step *t* to transfer to the next state $s_{t+1}^{n}$ based on the temporal observation $s_{t}^{n,T}$ for a specific length of time, which can be defined as $F\left(s_{t+1}^{n}|s_{t}^{n,T},a_{t}^{n}\right):S^{n,T}\times A^{n}\to P\left(s_{t+1}\right)$. Specifically, *F* is determined by the characteristics of the simulation software that implements the CAV environment. *R* denotes a reward function used to evaluate the performance of actions taken by vehicles in the current states. In addition, *R* is formulated according to the driving task requirements and vehicle optimization goals under different CAV driving environments. γ∈(0,1] is a discount factor for future reward, which is defined based on the expected weights of future reward values in the current CAV environment. The above parameters of TGMDP will be described in detail in the following part.

At a specific time step *t*, the current state of mixed autonomy traffic $S_{t}$ is extracted through graph representation to generate graphic features $G_{t}$. The GNN module uses the graphic features as input and generates processed features $Z_{t}$, which are then fed to the DRL module. Next, policy $\pi_{t}$ is produced by the DRL module, and a set of actions $a_{t}$ is generated to update the state of the traffic scenario state. Finally, the reward $R_{t}$ of the current time step is fed back to the GRL module to update model parameters.

It is important that each module in this proposed GRL framework can be adjusted and improved according to the actual need of the researcher. Specifically, the graph representation methods can be adjusted according to the traffic scenario characteristics and modeling approaches. The GNN and DRL modules in the GRL module can be freely substituted according to the actual need to achieve different combinations. The proposed framework can also be adjusted to different traffic environments.

### 6.2. Fundamental State Quantities and Data Flows of the GRL Framework

This section introduces the fundamental state quantities and data flows of the GRL framework in detail based on the proposed TGMDP.

#### 6.2.1. Temporal State Space $S^{n,T}$

In the proposed framework, the decision-making process of CAVs should not only consider the state at the current moment but also consider the set of environmental features in the previous time sequence of an appropriate length, to ensure the generation of reasonable driving behavior while minimizing the impact on the decision-making in real-time. Therefore, temporal state space $S^{n,T}$ is utilized, where *n* is the number of controlled CAVs participating in the decision-making task and *T* is the length of the observation time sequence.

#### 6.2.2. Temporal Graphic Feature $G^{n,T}$

For the decision-making task of CAVs, the aforementioned temporal state space needs to be mapped into feature vectors suitable as inputs for driving policy through a specific representation method. Considering the adaptability to the temporal features and the mutual effect between different vehicles, the graph representation method is carried out as the main mapping method for the feature vector generation in the proposed framework. Specifically, the temporal state space $S^{n,T}$ can be characterized as graph feature $G^{n,T}$. Taking any time step of the time sequence under consideration, $G^{n,T}$ can be further decoupled into node feature matrix $N\in R^{n\times f}$, which represents the set of vehicle eigenvectors in the environment, and adjacency matrix $P={\left(a_{ij}\right)}_{n\times n}\in \;R^{n\times n}$, which models the interaction between vehicles.

#### 6.2.3. Driving Policy π and Action Set $A^{n}$

Driving policy is a mapping function between the selection probabilities from the temporal state space $S^{n,T}$ to the action set $A^{n}$. If the CAVs select the strategy π at time step *t*, then the driving policy can be further written as $\pi (a_{t}^{n}|s_{t}^{n,T})$, which denotes the probability of the CAVs selecting the action set $a_{t}^{n}$. Specifically, the driving policy in the proposed GRL framework is the numerical solution of the temporal graph feature $G^{n,T}$ outputted by the GNN and the DRL in the GRL module. The driving policy π includes different types of action-value vectors, action probability density functions, etc., which are to be matched with the action space supported by the DRL algorithm.

The action set $A^{n}$ consists of behaviors that can be allowed by the decision-making system, with the specific actions selected based on the driving policy π. The action set can be represented as action space, which can be divided into discrete action space and continuous action space. High-level behaviors can only be represented as a discrete action space, whereas low-level control commands can be represented as a discrete action space. Different policy-generated methods of the DRL module generate different action spaces, which in turn generate different categories of driving behavior. The discrete action space is composed of a finite number of actions, which is typically the entire set of action commands available for a given task. For instance, in a lane-change scenario, the discrete action space can be defined as a=[changetoleft,gostraight,changetoright]. The discrete action space is encoded using one-hot vectors, where each encoded point corresponds to an action command, and all encodings are mutually incompatible. The continuous action space consists of specific values of control commands. For instance, in a highway scenario, the continuous action space can be defined as $a=[a_{t},\theta_{t}]$, where $a_{t}$ denotes the longitudinal acceleration and $\theta_{t}$ denotes the steering angle. The continuous action space is encoded using a multi-dimensional (or one-dimensional) vector, where each encoded position represents a control command. The control commands are normally limited to a certain value range, and specific values of the control commands are determined based on the adopted control strategy. The continuous action space can be discretized at a certain granularity, but in this case, the trade-off between the control accuracy and the action space dimension has to be considered.

In summary, the selection and generation of the action set $A^{n}$ should be matched with the driving policy π, and further correspond to the categories of policy-generated methods of the DRL module. The correspondence between the action set and the DRL methods is described in detail in Table 10.

#### 6.2.4. Reward Function *R*

In the TGMDP proposed in this paper, the reward function is to evaluate the performance of CAVs at a particular time step after they adopt a set of action strategies $A^{n}$ based on the environmental observations of the previous time sequence, which can be notated as $R(s_{t}^{n,T},a_{t}^{n})$. The reward function is an important part of the decision-making framework, and the comprehensiveness and reasonableness of its construction directly affect the final training effect of the algorithm. The reward function is usually formulated according to the driving task requirements and vehicle optimization goals under different driving scenarios and takes into account the trade-off between the overall and individual rewards of CAVs.

#### 6.2.5. Discount Factor γ

The discount factor is a hyperparameter in the TGMDP, and its specific value range is γ∈(0,1]. The function of the discount factor is to assess the importance of the current rewards and the future rewards obtained after taking a specific action set in the decision-making process for updating the parameters of the decision-making model. The larger the value of the discount factor, the more attention is paid to future rewards; otherwise, more attention is paid to current rewards. In addition, the discount factor allows for easy computation and avoids the infinite rewards that arise from an infinite MDP.

#### 6.2.6. Data Flow

The data flow is described based on the interaction trajectory between CAVs and the mixed autonomy traffic in TGMDP. At a specific time step *t*, the mixed autonomy traffic environment gives the temporal state space $S_{t}^{n,T}$ in a sequence of time periods from the time step *t* forward, which treated as the decision-making system’s observation of the driving environment. $S_{t}^{n,T}$ is then inputted to the graph representation module to perform feature extraction and generate the temporal graph features $G^{n,T}$. $G^{n,T}$ is fed to the GNN in the GRL module to generate decoupled graph features, which are further passed to the DRL module for processing through the policy network to generate the driving policy $\pi_{t}$ at the current time step *t*. Finally, the driving behavior module generates action set $A^{n}$ according to the driving policy π, which is fed back into the mixed autonomy traffic to control the motion of CAVs. The state of the driving environment is updated to the next time step $s_{t+1}^{n}$; at the same time, the mixed autonomy traffic evaluates the behavior at the current time step based on the designed reward function, calculates the reward value $R_{t}$ and feeds it back to the GRL module for updating the network parameters and optimizing the driving policies, thus realizing the complete cycle of data flow from the input of the temporal state space to the output of the action set based on the current driving policy. The overall function of the process can be represented by the following equation:

(4) $$A_{t}^{n}=\pi_{t}\left\{\Phi_{DRL,t}\left[\Phi_{GNN,t}\left(s_{t}^{n,T}\to g_{t}^{n,T}\right)\right]\right\}$$

where $\Phi_{GNN,t}$ denotes the graph convolution operator of the GNN module at time step *t* and $\Phi_{DRL,t}$ is the policy network mapping function of the DRL module at time step *t*.

### 6.3. Optimization Principle of the GRL Framework

After clarifying the basic principle and data flow of the framework, the driving policy optimization principle needs to be further specified to lay a theoretical foundation for the solving of the optimal driving policy of CAVs. The mapping from $s_{t}^{n,T}$ to $a_{t}^{n}$ to the feedback of the reward $R_{t}$ and the updating of the scenario state $s_{t+1}^{n}$ at each time step *t* can be defined as a trajectory $\left(s_{t}^{n,T},a_{t}^{n},R_{t},s_{t+1}^{n}\right)$ of the TGMDP. During the exploration of the interaction between the CAVs and the mixed autonomy traffic, many interaction trajectories are generated. By recording these trajectories, sampling them, and updating the parameters of the current decision-making policy network according to the reward values, the solution of the optimal driving policy relies on this process to iterate until the policy network parameters converge.

To explore the optimization principle, it is essential to define the optimization objective of driving policy for CAVs. A function that can characterize the optimization objective needs to be defined, and then this function needs to be approximated and optimized to continuously solve for the optimal driving policy. Generally speaking, the optimization objective of the driving policy is to ensure that individual CAV can receive the maximum combined reward for completing a particular driving task. Thus, the value function is introduced to assess the current decision-making system of CAVs in a given state. Value function can be divided into an action-value function and state-value function. In the proposed TGMDP, the action-value function refers to the expected discount return of the decision-making system of CAVs at the current time step *t*, with $\forall s_{t}^{n,T}\in S^{n,T}$ as the observed state, and $a_{t}^{n}\in A^{n}$ as the initial action, which is specified as:

(5) $$\begin{array}{cc} V_{\pi }^{Q}\left(s_{t}^{n,T},a_{t}^{n}\right) & =E_{\pi }\left[U_{t}|S_{t}^{n,T}=s_{t}^{n,T},A_{t}^{n}=a_{t}^{n}\right]\\ & =E_{\pi }\left[\sum_{i=0}^{\infty }\gamma^{i}U_{t+i}|S_{t}^{n,T}=s_{t}^{n,T},A_{t}^{n}=a_{t}^{n}\right] \end{array}$$

where $V_{\pi }^{Q}\left(s_{t}^{n,T},a_{t}^{n}\right)$ denotes the action-value function and $U_{t}$ denotes the discount return that describes the sum of the reward values of the decision-making system of CAVs and the accumulation of the reward values at the future moment with consideration of the discount factor γ. Considering that there are usually different action sets that can be chosen from for a state at a given time step, how to directly judge the value of the current state is also crucial to the optimization process of the driving policy, so the state-value function can be further introduced:

(6) $$\begin{array}{cc} V_{\pi }^{S}\left(s_{t}^{n,T}\right) & =E_{A_{t}^{n}\;\pi \left(\cdot |s_{t}^{n,T}\right)}\left[V_{\pi }^{Q}\left(s_{t}^{n,T},A_{t}^{n}\right)\right]\\ & =\sum_{a_{t}^{n}\in A_{t}^{n}}\pi \left(a_{t}^{n}|s_{t}^{n,T}\right)\cdot V_{\pi }^{Q}\left(s_{t}^{n,T},a_{t}^{n}\right) \end{array}$$

In this formula, the essential difference between the state-value function and the action-value function is to regard the action set $A_{t}^{n}$ that can be taken by the CAVs at the current time step as a random variable, and then solve the expectation of $A_{t}^{n}$ in order to eliminate the influence of $A_{t}^{n}$ on the value function. The obtained state-value function is only related to the time sequence of the current time step to measure the value of the decision-making system of CAVs in the current temporal state $s_{t}^{n,T}$. By unifying the action-value function and the state-value function to be denoted as *V*, the objective of the driving policy for CAVs can be further specified as follows:

(7) $$\max_{\pi }J_{\pi }=E_{\pi }\left[V_{\pi }\right]$$

Thus, the key to solving the optimal driving policy is to find the exact expression of the value function $V_{\pi }$ and to design different methods to maximize the value of $V_{\pi }$ in order to determine the state-action function of CAVs so as to determine the final optimal driving policy.

## 7. Validation for GRL-Based Decision-Making of CAVs

After proposing the methods for GRL-based decision-making of CAVs, it is essential to choose an appropriate way to evaluate the performance of the designed methods. This section summarizes the evaluation metrics that can be used with the GRL-based methods based on existing research, as well as relevant simulation tools that can be used to simulate and test the decision-making of CAVs.

### 7.1. Evaluation Metrics

Evaluation metrics are an important criterion for assessing the performance of methods. The relevant evaluation metrics that can be utilized to evaluate the decision-making performance are categorized in the following sections.

#### 7.1.1. Overall Evaluation

It is essential to evaluate the comprehensive performance of the proposed methods first. The reward function is a direct way that can well evaluate the performance of the designed methods generally.

- **Reward function:** The reward function directly affects the training process of GRL-based methods as well as the overall performance of decision-making. The design of the reward function needs to consider multi-dimensional evaluation metrics, and a reasonably designed reward function is crucial to the efficiency of vehicle decision-making. For the operation of CAVs, the establishment of a reward function should not only assess the overall decision-making performance of all controlled vehicles in the environment but also consider the individual performance of each vehicle.

#### 7.1.2. Dynamic Evaluation

The dynamic metrics assess the operational efficiency of the vehicle during its travel task.

- **Speed:** Speed is a direct way to assess the driving efficiency of the vehicle. In general, the maximum speed and average speed are usually chosen in related research [129].
- **Acceleration:** The maximum and average acceleration is typically selected to evaluate whether the vehicle can efficiently achieve high operating efficiency.

#### 7.1.3. Task Evaluation

A vehicle is necessary to complete a specified task during its operation process. The task metrics are used to assess the completion of the predetermined task of the vehicle.

- **Success rate:** This refers to the percentage of the driving task that the vehicles complete during the numerous training or testing episodes [96].
- **Finishing time:** This indicates the time it would take for the vehicle to complete its driving task [130].
- **Iterative steps:** In some simulation platforms, the system returns the number of steps consumed when the vehicle completes the driving task. Therefore, the iteration step can be used as an indicator to assess the efficiency of task completion [40].

#### 7.1.4. Safety Evaluation

Safety is the most important guarantee for the efficient operation of vehicles. Safety evaluation is an essential part in designing decision-making methods.

- **Number of collision:** This refers to the collision number between vehicles in each training or testing episode [131].
- **Time to collision:** This refers to the time of collision between the ego-vehicle and the front vehicle, which can be calculated from the relative distance and relative speed [132].
- **Number of lane changes:** This implies a trade-off between safety and efficiency in vehicle operation. If lane changes are too frequent, vehicles are prone to accidents; conversely, high driving efficiency may not be guaranteed [133].
- **Vehicle jerk:** This represents the urgency of the longitudinal or lateral control of the vehicle [134].
- **Traffic rules:** Obeying traffic rules is an essential part of safe driving. This metric mainly refers to whether the vehicle violates traffic rules during operation and the frequency of violations. Compliance with traffic rules requires an assessment of whether the controlled vehicle has run a red light, invaded the lane mark, driven out of the road, driven on the wrong line, blocked the future path of other vehicles, etc. [96,135,136].

#### 7.1.5. Economy Evaluation

Economic metrics are used to evaluate the driving economy of vehicles.

- **Energy consumption:** Energy consumption is an important indicator for assessing vehicle economy. It can be divided into fuel consumption and electricity consumption depending on the type of power used in the vehicle [99,100,101].

### 7.2. Relevant Simulation Tools

Choosing an appropriate simulation tool is important to promote the approach. This section summarizes the feasible open-source simulation platforms and program libraries that can help to carry out the research on GRL-based decision-making systems. A summary of the simulation tools is presented in Table 11.

### 7.3. Initial Test of GRL-Based Methods

**Figure 6:**
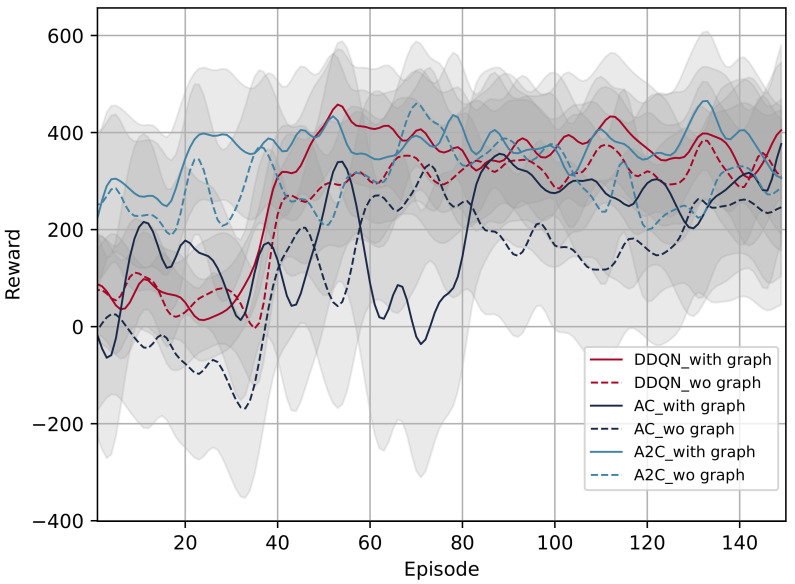
The curves of the average training reward of several GRL-based methods and their corresponding DRL-based methods. The shaded areas show the standard deviation for three random seeds; the solid line represents the reward curve of a GRL-based method, while a dashed line represents the reward curve of a DRL-based method.

For preliminary verification, the optimization effect advantage of the GRL-based methods relative to the DRL-based methods, this section conducts an initial test based on our previous research [40]. A GRL-based decision-making model is trained in a highway ramping scenario. The ablation experiment was conducted to verify the effectiveness of the GRL-based methods. Double DQN, AC, and A2C were chosen as the main test algorithms. Three random seeds were trained for 150 epochs, and the average training reward was calculated as the evaluation of a method. The performance of different GRL-based methods and their corresponding DRL-based methods are summarized in Table 12. The reward curves of several GRL-based methods in the two constructed scenarios are illustrated in Figure 6.

The experimental results showed that all GRL-based methods had higher average rewards than their corresponding DRL-based methods in both test scenarios. In addition, the curves of the average training reward of GRL-based methods are above that of DRL-based methods in general. The above initial results indicated that implementing graphic techniques into the DRL methods could improve the multi-agent decision-making performance of CAVs in mixed autonomy traffic.

## 8. Challenges and Future Outlook

This section identifies the current challenges and presents future research directions in the field of GRL-based methods for decision-making in mixed autonomy traffic based on the results of the state-of-the-art research and comparative study presented in this paper. In addition, each future research issue is explained with a schematic to increase the readability for readers.

### 8.1. Communication

**Figure 7:**
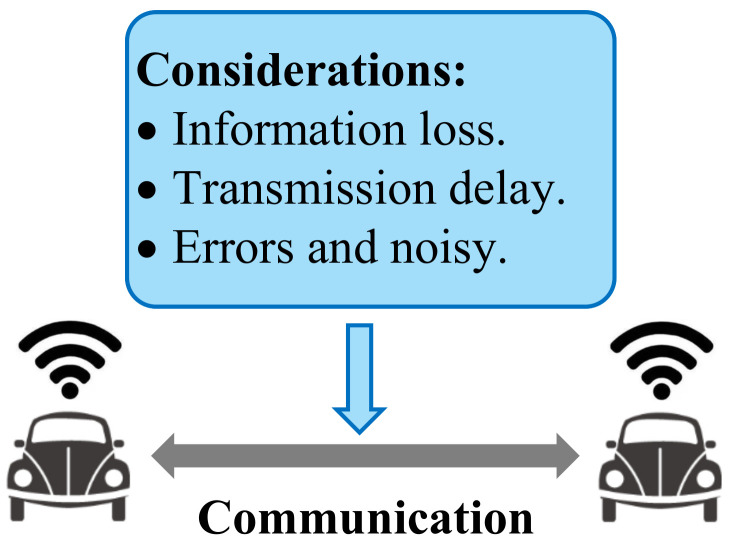
Description of the future perspectives for communication research.

The data sharing among CAVs at the perceptual information level and their collaboration at the motion planning level rely on efficient communication between vehicles and data transmission between vehicles and centralized controllers. In [137], the authors discussed the influence of packet loss on information transmission in distributed vehicle control problems. In [138], the impact on collaborative multi-agent decision-making in communication failure scenarios was investigated. With the development of 5G technology, low-power and low-latency technologies have significantly developed. Furthermore, in [139], it was investigated how to model communication mechanisms in intelligent transportation systems using GNNs. The model constructed in this paper does not consider the effects of information loss, errors, and delays in vehicle communication on the results. In [140], the authors modeled the effect of information communication and delay on real-world multi-robot systems using the graph structure. The structured modeling with a GNN facilitates the simulation and processing under more realistic future traffic conditions. A future direction regarding the proposed GRL-based framework could be to model the effect of communication between vehicles in the collaborative decision-making model. The schematic of this issue is elaborated on in Figure 7.

### 8.2. Reward Design

**Figure 8:**
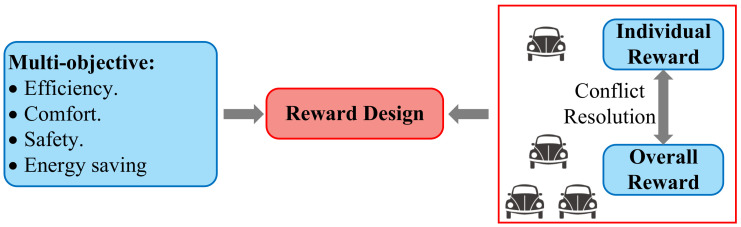
Description of future research for reward design.

In the introduced decision-making model based on DRL, the final model performance depends on the reward function design in the DRL module and the weight distribution of the rewards [7,22]. Therefore, an implementation strategy of the requirements for model performance into the designed reward functions can significantly affect the training results [141]. For instance, in the considered highway ramping and Figure-Eight scenarios, the definition of reward was influenced by the scenario and task performances (e.g., the overall traffic efficiency, traffic efficiency in a particular lane, and reduction in the passage time of a specific type of vehicle in a scenario). Moreover, for the cooperative multi-agent decision-making problem in the mixed autonomy traffic, the conflict between the overall reward and the individual reward must be considered. This includes social interaction and implicit synergy between human drivers with different levels of aggressiveness [7]. The design process of the reward function also needs to consider the priority of HVs and AVs, and such priorities need to be taken into account in the design of the loss function, with the development and robustness of laws and regulations involving autonomous driving. The schematic of this issue is elaborated on in Figure 8.

### 8.3. Transfer Learning

**Figure 9:**
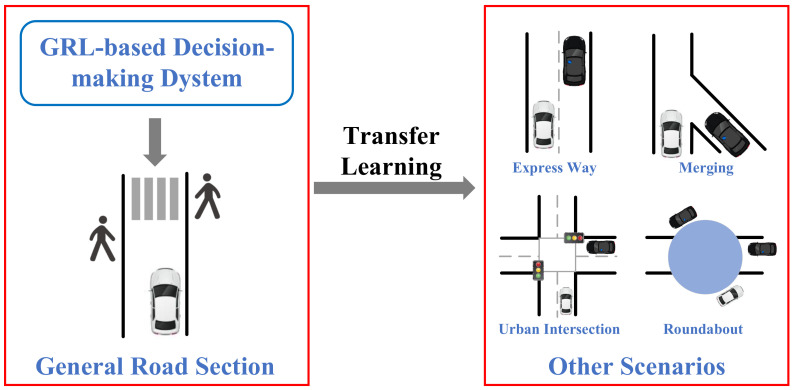
Description of future research for transfer learning.

The reviewed approaches and the proposed framework focus on the multi-agent collaborative approach based on RL. According to [142], when the distribution of scenarios where models are trained and test scenarios have certain differences, the performance of the RL-based models decreases. To solve the migration learning problem involved in model adaptation to unseen traffic scenarios, the constructed models need to be adapted to the new scenarios encountered in tests. However, this can degrade model performance in historical scenarios learned during the training process, which is referred to as catastrophic forgetting. Therefore, one of the current work challenges is making the models capable of continuous learning and evolution. Moreover, the designed models need to be stable, incremental, and efficient in adapting to new scenarios and environments following the changes in a model’s test and actual traffic scenarios [143]. The schematic of this issue is elaborated on in Figure 9.

### 8.4. Human Factor

**Figure 10:**
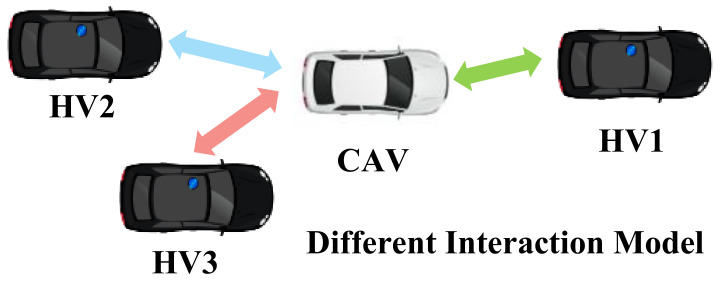
Description of future research that considers human factors.

According to [144], human drivers and passengers in AVs have different levels of risk acceptance and perception. Current research has not adequately considered the human perceived risk for driver and passenger comfort. In [145], it was pointed out that the risk range around a vehicle should be a risk field related to the driver’s risk acceptance level. Therefore, the developed model needs to consider the individualized risk acceptance levels. Meanwhile, in complex and intense interaction environments and scenarios, the interactions between human drivers and between self-driving vehicles and HVs are different, so the variability brought by the human factor should be considered. The schematic of this issue is elaborated on in Figure 10.

### 8.5. Traffic Control System Cooperative Feature

**Figure 11:**
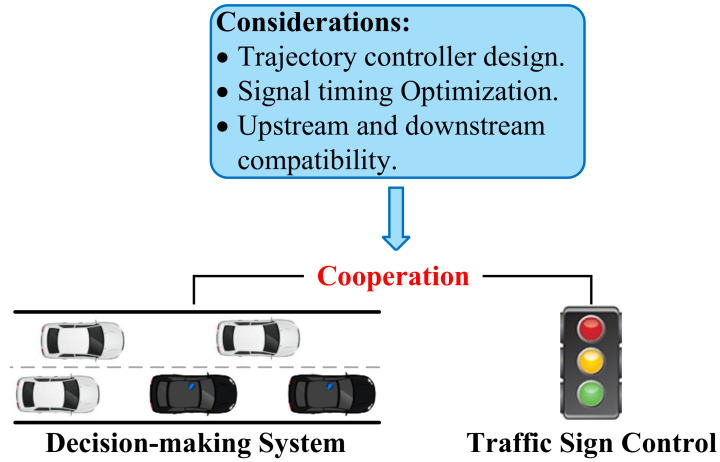
Future research points for traffic control system cooperation.

In this study, traffic signals, which are widely available in urban road networks, have not been modeled in the current phase of the model. However, in future intelligent transportation systems, the optimization of vehicle decision control behavior and traffic signal control phases should be a coupled model. In the case of complete vehicle-road information, CAVs on urban roads can accurately sense and predict the signal timing scheme of downstream intersections and adjust the trajectory accordingly. The signals can also actively provide real-time information on the position and speed of arriving vehicles and optimize the signal parameters. Thus, fleet trajectory control and traffic signal optimization are interdependent and affect each other. Namely, the trajectory control or signal optimization alone cannot significantly improve the intersection capacity and traffic flow operation efficiency. Only collaborative optimization of intelligent, networked fleet trajectory and traffic signal can achieve the goals of minimum delay, stop times, fuel consumption, emissions, and optimal traffic efficiency [146]. Therefore, future research should focus on the three following points: (1) how to design the trajectory control algorithm and strategy for the intelligent networked fleet so that vehicles can slow down smoothly in the face of red-light signals to achieve the minimum number of stops, fuel consumption, and emissions; (2) how to make full use of fleet information to optimize the signal timing scheme to achieve the control objectives of minimum delay and optimal traffic efficiency; (3) how to be compatible with upstream and downstream intersections to extend the optimization control to the road network and solve the optimization problem in real time. The schematic of this issue is elaborated on in Figure 11.

### 8.6. Uncertainty Problem

**Figure 12:**
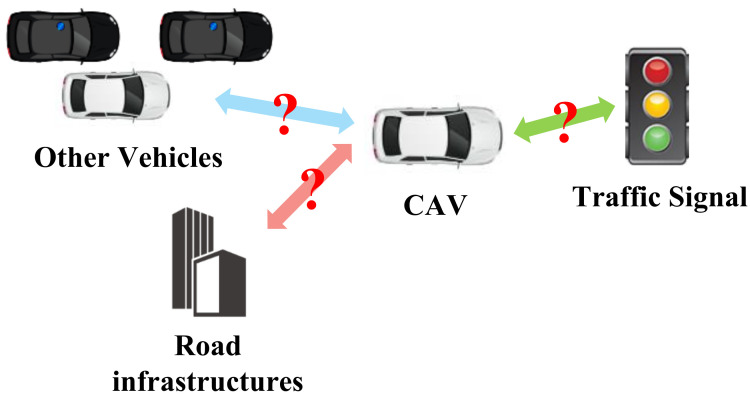
Description of the uncertain problem in mixed autonomy traffic.

The performance of machine-learning-based models greatly depends on the training data selection, which can result in accidental and cognitive uncertainties. The accidental uncertainty (non-reducible) is caused by the inherent randomness in the collected data, which includes the imperfection of information sharing in mixed autonomy traffic and sensor shift [147]. For instance, in a lane-changing scenario, AVs may not be able to obtain the turn signal of the surrounding HVs accurately, which will result in a decrease in the passable area, which in turn affects the decision-making process of AVs in a traffic environment. Cognitive uncertainty, which is also known as knowledge uncertainty, originates from the rare cases in the test environment, which denote cases that rarely occur in the training environment; this problem can be addressed by increasing the amount of training data. The proposed model does not systematically consider the effects of the two uncertainties on the model performance. Furthermore, in this work, it has not been analyzed how to reduce the cognitive uncertainty and adapt to the new test environment through the model structure adjustment. In future work, a multi-agent GRL-based model for the mixed autonomy traffic considering uncertainty could be considered. The schematic of this issue is elaborated on in Figure 12.

### 8.7. Coordination of Global and Local Information

**Figure 13:**
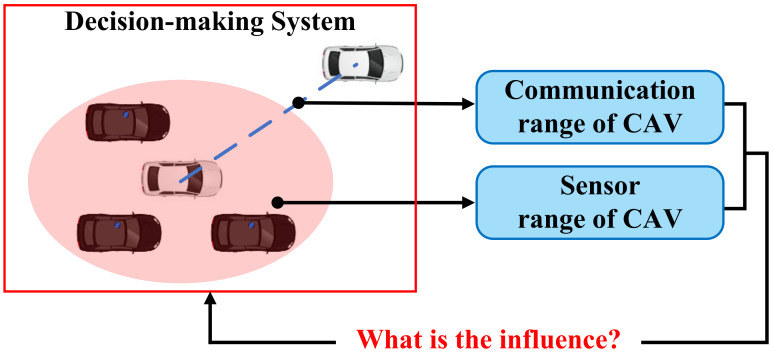
Description of the influence of global and local information coordination on the decision-making system.

The model constructed in this study assumes that there is communication between all AVs and that the sensing ability of a sensor is within a defined perception range. In the graph structure design, communication between vehicles and sensing range can be reflected by the connectivity between nodes in a graph. In future work, the impact of the perception range and communication range of AVs on the overall performance of the model could be analyzed based on a GRL-based framework. In addition, it could be discussed whether the requirement for the sensing range of a single AV could be decreased to reduce the sensor cost and avoid the uncertainty problem in remote sensing. The schematic of this issue is elaborated on in Figure 13.

### 8.8. Vehicle Models

**Figure 14:**
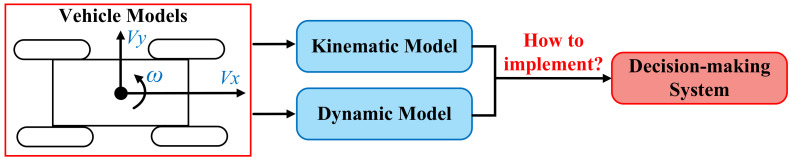
Future research of the vehicle models.

The proposed GRL model for decision-making in this paper uses a simplified vehicle kinematics model configured by [1]. Accordingly, the action space of RL considers only several discrete decision behaviors, including lane-changing commands. However, in this work, the whole lane-changing process is represented by an ideal model. Still, more complex vehicle kinematics and dynamics models should be considered in the future, since the road conditions and the parameters of a vehicle model are crucial for an accurate evaluation of vehicle motion. The schematic of this issue is elaborated on in Figure 14.

## 9. Conclusions

This paper reviews the GRL-based methods for multi-agent decision-making in mixed autonomy traffic. Firstly, a generic and modular GRL-based framework is proposed, and the techniques of each module in the proposed framework are elaborated. Then, a review of GRL-based methods for solving decision-making problems in mixed autonomy traffic is provided based on the different modules in the proposed framework. In addition, some available validation approaches are summarized to provide an efficient way to verify the designed GRL-based decision-making methods. Finally, the current challenges and future research directions in the field of GRL-based methods are forecasted. This work has great potential to provide researchers with adequate reference in the design of GRL-based methods for decision-making in mixed autonomy traffic. However, there is still a lot of work to be done on the design of the decision-making system for CAVs, and the performance of the decision-making methods in real-world environments urgently needs to be tested in future research.

## Figures and Tables

**Table 2:** Summary of surveys on decision-making and RL technology for autonomous driving.

Refs.	Topic	Year	Range of Discussions				Characteristic
			**Framework**	**RL**	**DRL**	**GRL**	
[27]	decision-making	2018	✕	✕	✕	✕	Rule-based methods and application were mainly discussed.
[28]	Planning; decision-making	2018	✕	✓	✕	✕	A wide range of categories of decision-making approaches were summarized.
[23]	decision-making	2021	✓	✓	✓	✕	A framework was proposed, and several categories of methods were summarized.
[24]	DRL in autonomous driving	2020	✕	✓	✓	✕	DRL-based applications in several research fields of autonomous vehicles were presented.
[25]	DRL in ITS	2021	✕	✓	✓	✕	DRL-based approaches for ITS, as well as the principle of DRL algorithm in ITC were mainly discussed.
[26]	GRL in different fields	2022	✕	✕	✓	✓	Typical GRL-based algorithms and applications in several fields were generally summarized.
[29]	GRL in different fields	2022	✓	✕	✕	✕	Basic knowledge and general technology roadmap of CAVs were mainly summarized.
**Ours**	GRL in decison-making	2023	✓	✓	✓	✓	A comprehensive review of GRL-based methods for decision-making systems of CAVs were presented, including framework, related research, and validation methods.

**Table 5:** Summary of the typical GNN algorithms that can be used for GRL-based decision-making.

Category	Algorithm	Refs.	Characteristic	Pros and Cons
**Convolutional-** **Based**	GCN	[49]	Classical GNN method implemented from the spectral domain. Fourier transform is utilized to apply Laplace matrices to graph structure data. The entire graph is needed for learning the node representation, resulting in low efficiency.	✓ Easy to be implemented and derived. ✓ Global information of the graph structure data can be well captured. - Difficulty in large-scale graph data. - Poor flexibility and very poor scalability. - Slow convergence rate.
	GraphSAGE	[50]	Extending traditional GCN method to the spatial domain to large-scale graphic data. Neighboring nodes are locally sampled to increase computing efficiency. Different aggregation functions can be used to couple the features of the neighboring nodes. Can be applied to large-scale graphic structures.	
	GWNN	[51]	An improved spectral domain approach based on typical GCN. Graph wavelet transform is adopted to avoid the high computational matrix decomposition.	
	LADIES	[52]	An improved spatial domain approach based on typical GCN. Hierarchical importance sampling is utilized to reduce memory usage.	
**Attention-** **Based**	GAT	[53]	The typical approach of introducing attention mechanisms into GNN from the spatial domain. Relative weights between connected nodes can be learned. High computational efficiency and feature utilization.	✓ Better characterisation of interactions. ✓ More pertinent feature extraction. - Over-smoothing usually occurs. - Underutilization of edge information.
	SAGNN	[54]	A self-attentive GNN is proposed based on the GAT method. Feature learning and graphic data processing of heterogeneous graphs can be initially performed.	
	SpGAT	[55]	The first migration of the attention mechanism to the spectral domain based on the GAT method. The graph is decomposed into low and high-frequency components with two convolution kernels. The global and local information of the graph can be effectively encoded.	
**Spatial-Temporal-** **Based**	DynamicGCN	[56]	The LSTM is implemented into typical GCN methods. The features of GCNs from different time slices in dynamic networks can be captured.	✓ High efficiency of feature extraction. ✓ Simultaneous access to spatial and temporal information. - High computational cost. - Complexity of graphic structuremodeling.
	ST-GCNN	[57]	A typical method that incorporates the temporal convolutional network (TCN) into GCN. Sequential graph-structure data can be effectively handled.	
	Social- STGCNN	[58]	A Time-Extrapolator Convolution Neural Network (TXP-CNN) is proposed based on ST-GCNN. Graph embedding originating from ST-GCNN can be utilized to solve prediction tasks.	

**Table 7:** Summary of the Characteristic of Typical DRL Algorithms.

Category	Algorithm	Refs.	Available Scenario		Characteristic	Pros and Cons
			**Discrete**	**Continuous**		
**Value-Based**	DQN	[71]	✓	✕	Neural network is introduced to approximate action-value function. A target Q network is used to generate and update the target Q-values Experience replay is applied to break down the correlation between samples. Suitable for large state spaces. Suffering from the overestimation of action values under certain conditions.	✓ Suitable for high-level decision-making. ✓ Wide range of applications. - Difficult to apply to stochastic policies and continuous action spaces. - Slow convergence. - Over estimation.
	Double DQN	[72]	✓	✕	The selection of the action is decoupled from the evaluation of the target Q value. Significantly mitigate bias caused by bootstrapping.	
	Dueling DQN	[73]	✓	✕	The action-value function is decoupled into a state-value function and optimal advantage function. More accurate estimation of action-value functions. The convergence process is accelerated. Overestimation of action value still exists.	
	Noisy DQN	[74]	✓	✕	Add noisy function to the parameters of the neural network. Strong robustness. Provide a broader space for action exploration. Large computational cost.	
	DQN with PER	[75]	✓	✕	Assign different priorities to the samples in the replay buffer. High sample utilization. High learning efficiency. The learning rate needs to be adjusted reasonably.	
	Distributional DQN	[76]	✓	✕	The DRL formulation is modeled from a distributional perspective. The histogram is chosen to represent the estimate of the value distribution. More accurate risk assessment of different actions.	
	Rainbow DQN	[77]	✓	✕	Integrate all the previous DQN-based methods. Multi-step learning is utilized to accelerate learning speed.	
**Policy-Based**	REINFORCE	[78]	✓	✓	The typical Monte Carlo-based policy gradient algorithm. Stochastic gradient ascent is used to update the model parameter. Large variance of gradient estimation. Poor model stability and low learning efficiency.	✓ Better convergence. ✓ Suitable for high-dimensional continuous action spaces and stochastic policy. - Easily converges to a non-optimal solution. - Inability to make full use of historical state.
	AC	[79]	✓	✓	The DRL model consists of an actor network and a critic network. Actor network predicts the probability of the action, critic network predicts the value in the current state. Single-step update can be performed to achieve high update speed. Correlation exists between parameter updates of the two neural networks. Difficult model convergence since the action generated by the actor network depends on the value predicted by the critic network	
	A2C	[80]	✓	✓	Add a baseline to the calculation of Q values based on AC method. Reduce numerical variation in the actor networks. Strong model stability.	
	NAF	[81]	✕	✓	Normalized advantage function is designed to extend DQN method to continuous action space. Q value is decoupled into value function and advantage function, and advantage function is calculated based on the Cholesky decomposition.	
	DDPG	[82]	✕	✓	The actor-critic framework is introduced based on the DQN method. Target networks are created for both actor and critic networks. Experience replay is established to ensure high sample efficiency. Deterministic policy is not conducive to action exploration. Overestimation of Q value generated by the critic network.	
	TD3	[83]	✕	✓	Double Q learning is implemented based on the DDPG method to reduce the overestimation of Q value Delay actor network updates for more stable training for actor network. Add noise to the output action from the target actor network to increase the stability.	
	PPO	[84]	✓	✓	Importance sampling is applied to change the actor-critic framework from an on-policy to an off-policy scenario. A clipped surrogate objective is proposed to limit the amplitude of policy updates to avoid excessive strategy deviations. Multiple training updates with one sample. Good versatility and sample complexity.	

**Table 10:** Calculation Methods of driving behaviors using different DRL methods.

Categories	Driving Policy	Derivation of Driving Behaviors
Value-based	State-value function	**Discrete**: Value of each available action.
Policy-based	Deterministic policy	**Continuous**: Specific numerical instruction of each action.
	Stochastic policy	**Discrete**: Probability of each available action.
		**Continuous**: Normal distribution of each available action.

**Table 11:** Summary of the simulation tools that can be used for GRL-based decision-making.

Category	Name	Discription	Support Language	Link
Simulation platform	Carla	Realistic simulation scenarios with different sensor models; focus on simulation of environmental perception system; support for RL-based decision-making; support for complex vehicle control algorithms.	Python	https://github.com/carla-simulator/carla (accessed on 8 December 2022)
	SUMO	Macro-scale modeling of traffic scenario; multi-agent decision-making is well supported; python interface is achieved by implemented TRACI.	Python/C++	https://www.eclipse.org/sumo/ (accessed on 15 June 2023)
	FLOW	A DRL-based framework that provides integration with DRL library and traffic micro-simulation platform; several traffic control benchmarking are presented.	Python	https://flow-project.github.io/ (accessed on 18 March 2023)
	Open-CDA	Combining Carla and SUMO for Co-simulation; full-stack prototype cooperative driving system can be achieved.	Python	https://github.com/ucla-mobility/OpenCDA (accessed on 6 October 2022)
	Highway-env	A gym-based simulation environment for typical traffic scenario; good support for RL-based multi-agent decision-making	Python	https://github.com/Farama-Foundation/HighwayEnv (accessed on 19 March 2023)
	CommonRoad	Good simulation of planning and multi-agent decision-making of autonomous vehicles; comprehensive support for RL-based methods; provides numerous traffic scenarios for validation.	Python	https://commonroad.in.tum.de/ (accessed on 20 February 2023)
Program library	Pytorch	A popular machine learning tools with fast updating and comprehensive documentation.	Python/C++ /Java	https://pytorch.org/ (accessed on 9 July 2023)
	Tensorflow	A stable machine learning library with highly visualized and easy debugging.	Python/C++ /Java	https://www.tensorflow.org/ (accessed on 8 January 2023)
	Pytorch geometric	An easily write and train GNN library based on Pytorch for a wide range of applications.	Python	https://pytorch-geometric.readthedocs.io/en/latest/index.html (accessed on 5 June 2023)
	Tensorflow geometric	An efficient GNN library based on Tensorflow.	Python	https://github.com/CrawlScript/tf_geometric (accessed on 16 September 2022)
	RLlib	A production-level and highly distributed RL-based framework supporting both Pytorch and Tensorflow; unified and simple APIs for a large variety of industrial applications.	Python	https://docs.ray.io/en/latest/rllib/index.html (accessed on 12 January 2023)
	Stable Baseline3	A set of reliable implementations of RL-based algorithms in PyTorch; high frequency of continuous updates.	Python	https://github.com/DLR-RM/stable-baselines3 (accessed on 25 October 2022)
	Pfrl library	A library that implements various state-of-the-art DRL-based algorithms.	Python	https://github.com/pfnet/pfrl (accessed on 7 May 2023)

**Table 12:** Performance of different GRL-based methods.

Method	Average Reward of DRL	Average Reward of GRL	Optimization Rate (%)
Double DQN	337.79	374.51	10.87
AC	132.40	192.63	45.49
A2C	298.61	363.93	21.88

## Data Availability

Not applicable.
